# Glycochenodeoxycholic acid promotes hepatocarcinogenesis by inducing hepatic progenitor cell differentiation into cancer-associated fibroblasts via sphingosine-1-phosphate receptor 2 signalling

**DOI:** 10.1186/s40364-025-00873-0

**Published:** 2025-12-17

**Authors:** Lu Gao, Gang Lv, Ying Huang, Mengmeng Xue, Zhipeng Zhang, Jing Weng, Haoran Bai, Min Tao, Xi Luo, Yuhua Gao, Shiyao Feng, Xiaojuan Hou, Chen Zong, Xue Yang, Qiudong Zhao, Jinghua Jiang, Xinyu Zhu, Zhipeng Han, Changtao Jiang, Dongyu Zhao, Lixin Wei, Lulu Sun

**Affiliations:** 1 Department of Physiology and Pathophysiology, School of Basic Medical Sciences, State Key Laboratory of Vascular Homeostasis and Remodeling, Peking University, Beijing, China; 2 Department of Tumor Immune and Metabolism, National Center for Liver Cancer, Naval Medical University, Shanghai, China; 3 State Key Laboratory of Female Fertility Promotion, Department of Endocrinology and Metabolism, Third Hospital, Peking University, Beijing, China; 4 Senior Department of General Surgery, The First Medical Center of Chinese PLA General Hospital, Beijing, China; 5 Department of Biomedical Informatics, School of Basic Medical Sciences, Peking University, Beijing, China; 6 School of Pharmacy, Anhui Medical University, Hefei, China; 7 Institute of Advanced Clinical Medicine, Peking University, Beijing, China; 8 Department of General Surgery, Peking University Third Hospital, Peking University, Beijing, China; 9 Cancer Center, Beijing Key Laboratory for Interdisciplinary Research in Gastrointestinal Oncology, Peking University Third Hospital, Peking University, Beijing, China; 10 Department of Oncology, Longhua Hospital Affiliated to , Shanghai University of Traditional Chinese Medicine, Shanghai, China; 11 State Key Laboratory of Female Fertility Promotion, Center for Reproductive Medicine, Department of Obstetrics and Gynecology, Peking University Third Hospital, Beijing, China; 12 Department of Urology, Chao hu Hospital of Anhui Medical University, Anhui, China; 13 Changhai Clinical Research Unit, Changhai Hospital of Naval Medical University, Shanghai, China

**Keywords:** Hepatocellular carcinoma, Hepatic progenitor cells, Cancer-associated fibroblasts, Glycochenodeoxycholic acid, Sphingosine-1-phosphate receptor 2

## Abstract

**Background:**

Patients with advanced hepatocellular carcinoma (HCC) often develop cholestasis and exhibit poor clinical outcomes. Current evidence suggests that dysregulated bile acid metabolism may contribute to HCC progression, although the underlying molecular mechanisms remain unclear. Among the bile acids, glycochenodeoxycholic acid (GCDCA) is a key component of cholestasis. Under pathological conditions, hepatic progenitor cells (HPCs) transform into fibroblasts or tumor-initiating cells, directly promoting liver fibrosis and HCC development. This study aimed to investigate the regulatory role of GCDCA in the activation and differentiation of HPCs to elucidate its potential mechanisms in hepatocarcinogenesis.

**Methods:**

Single-cell RNA sequencing was used to infer the heterogeneity of cancer-associated fibroblasts (CAFs) and their differentiation relationship with HPCs in a diethylnitrosamine-induced rat model. The TCGA and GEO datasets were used to assess the prognostic value of inflammatory CAFs (iCAFs) in patients with HCC. Transcriptomic sequencing, functional assays, and in vivo experiments were performed to validate the role of GCDCA in HCC pathogenesis.

**Results:**

We identified a CAF subset closely associated with the development of HCC (PDGFRA^+^CAFs). GCDCA promotes the differentiation of HPCs into PDGFRA^+^CAFs, which is mediated by sphingosine-1-phosphate receptor 2 (S1PR2). Treatment with the S1PR2 inhibitor JTE-013 markedly inhibited the progression of the pro-fibrotic phenotype in HPCs, which consequently reduced tumor growth and fibrosis in rats.

**Conclusions:**

GCDCA-induced differentiation of HPCs into PDGFRA^+^CAFs plays a critical role in HCC progression, driven by the S1PR2 receptor. These results provide new insights into the mechanisms underlying HPCs-mediated hepatocarcinogenesis. Targeting S1PR2 represents a promising therapeutic strategy for HCC with potential benefits in reducing fibrosis and tumorigenesis.

**Supplementary Information:**

The online version contains supplementary material available at 10.1186/s40364-025-00873-0.

## Background

Cholestasis frequently coexists with chronic liver diseases and hepatocellular carcinoma (HCC). Both primary biliary cholangitis (PBC) and primary sclerosing cholangitis (PSC) are cholestatic liver diseases associated with a significantly increased risk of HCC [1, 2]. Patients with HCC and concurrent jaundice have a poor prognosis, with a median survival of only 2–4 months [3, 4]. These findings strongly suggest that dysregulation of bile acid (BA) metabolism is a key driver of HCC progression. However, effective targeted therapies for high-risk populations are lacking.

Recent studies have elucidated an association between HCC and aberrant differentiation of hepatic progenitor cells (HPCs). HPCs are oval-shaped cells located in the portal area that possess self-renewal, proliferation, and multipotent differentiation capacities [5–8]. In chronic liver disease, HPCs can abnormally differentiate, beyond their conventional differentiation into hepatocytes/cholangiocytes for tissue repair [7, 9], into either tumor-initiating cells or myofibroblasts [10–12]. Using cell transplantation and genetically engineered mouse models, Sekiya et al. first demonstrated that hepatic HPCs can differentiate into myofibroblasts, providing direct evidence of their involvement in fibrosis and tumorigenesis [10]. However, under conditions of cholestatic liver injury, the mechanisms by which HPCs respond to microenvironmental signals, the molecular pathways governing their fate decisions, and the functional relevance of this process in HCC initiation and progression remain unclear.

Cancer-associated fibroblasts (CAFs), the most abundant extracellular matrix components in the tumor microenvironment, exhibit high heterogeneity and plasticity [13–15]. Emerging evidence indicates that during hepatic fibrosis and tumor development, resident fibroblasts and other cell types undergo activation and differentiation into myofibroblasts or CAFs in response to various pathological stimuli [16]. CAFs play a crucial role within the tumor microenvironment (TME). CAFs have been shown to promote cancer cell proliferation, migration, invasion, and epithelial-mesenchymal transition (EMT) through paracrine signaling-mediated crosstalk [13, 17, 18]. Over the past decade, several CAFs-derived chemokines such as interleukin-6 (IL-6), C-X-C motif chemokine ligand 12 (CXCL12), and C-C motif chemokine ligand 2 (CCL2) have been implicated in the mechanisms driving tumor progression [13, 19, 20]. Therefore, as a key component of the TME, CAFs play a critical role in the initiation and progression of HCC [21, 22]. However, the complex interactions between fibroblasts, liver cancer cells, and HPCs are not yet fully understood.

Chronic cholestasis leads to characteristic changes in BA metabolism [23], with glycochenodeoxycholic acid (GCDCA) specifically elevated in patients with fibrosis and HCC [24, 25]. Studies have indicated that GCDCA contributes to the progression of liver diseases through various mechanisms, such as inducing lipid metabolic disorders and activating the nuclear factor kappa B (NF-κB) inflammatory cascade [26]. Our previous research in mouse models revealed that GCDCA-treated HCC cells exhibit enhanced metastatic potential [27]. Thus, GCDCA may act as a key driver linking chronic cholestasis, hepatic fibrosis, hepatocarcinogenesis, and ultimately tumor metastasis. GCDCA likely establishes a critical pathogenic axis driving the progression from chronic liver disease to HCC by disrupting metabolic homeostasis and activating pro-inflammatory and pro-fibrotic signaling pathways. Based on these findings, we propose the hypothesis that exposure to high levels of bile acids may promote the differentiation of HPCs into CAFs, thereby accelerating the malignant transition from liver fibrosis to HCC.

In our study, we aimed to investigate the regulatory role of GCDCA in the activation and differentiation of HPCs, with a focus on elucidating the mechanisms underlying their contribution to hepatocarcinogenesis, the heterogeneity of CAFs, and the potential differentiation relationship between CAFs and HPCs using single-cell RNA sequencing in a rat model.

## Methods

### Animals and experimental design

Male wild-type Sprague-Dawley rats (aged 8–10 weeks, 160–180 g) were obtained from Shanghai Ji Hui Laboratory Animal Care Co., Ltd. and housed in a specific pathogen-free facility. To induce hepatocarcinoma, rats were administered diethylnitrosamine (DEN) at a concentration of 100 p.p.m. (95 µg/mL) in drinking water. At week 8 of DEN induction, GCDCA treatment was initiated. Rats were anesthetized with pentobarbital and administered GCDCA (8 µmol/100 g body weight) or an equivalent volume of PBS twice weekly via intraperitoneal injection [28]. To reduce endogenous BA reabsorption, DEN-treated rats were fed a diet containing 1.2% cholestyramine. In addition, JTE-013 was intraperitoneally injected 1 h prior to GCDCA infusion at a dose of 2 mg/kg [29]. At week 12, the rats were euthanized, and biochemical tests were performed. Serum concentrations of alanine transaminase (ALT), aspartate transaminase (AST), and total BA (TBA) were measured. Liver tumor formation was assessed using electronic calipers and liver tissue samples were fixed in 10% neutral-buffered formalin for histopathological examination.

### Reagents

DEN, GCDCA, and cholestyramine were purchased from Sigma-Aldrich (St. Louis, MO, USA). JTE-013 was obtained from Taoshu Biology (Shanghai, China).

**Cell culture and conditioned medium collection**

WB-F344 cells (rat liver progenitor cell line) were maintained in Dulbecco’s Modified Eagle Medium (DMEM) supplemented with 10% fetal bovine serum (FBS) and 1% penicillin-streptomycin at 37 °C under 5% CO₂. Following a two-week treatment with 100 µmol/L GCDCA, morphological changes in HPCs were observed. To validate CAFs, the expression of the fibroblast marker α-SMA was detected using immunofluorescence staining. Conditioned media (CM) were prepared from the generated CAFs. After, the cells were washed by PBS three times and incubated in serum-free DMEM for 24 h, after which the supernatant was collected. Similarly, CM was collected from WB-F344 cells, which served as a control, using the same protocol. All collected media were centrifuged (1500 × g, 10 min, 4 °C) to pellet cellular debris, then aliquoted and stored at -80 °C. The CM derived from CAFs and WB-F344 cells are referred to as GCDCA-CM and PBS-CM, respectively.

### Cell transfection

HEK293T cells (Procell Life Science, China) were cultured in Dulbecco’s Modified Eagle Medium (DMEM) supplemented with 10% fetal bovine serum (FBS) and 1% penicillin-streptomycin at 37 °C under 5% CO₂. Transient transfection was performed using Lipo8000™ transfection reagent (Beyotime, China) according to the manufacturer’s protocol. Cell harvest was performed at specified times post-transfection.

### Establishment and cultivation of adult rat liver organoids

Primary hepatic progenitor cells were extracted from the rats previously treated with DEN. Following anesthesia under sterile conditions, the liver was surgically excised and maintained at 4 °C in basal medium in a 10 cm culture dish. The tissue was minced into approximately 0.5 mm^3^ fragments using scissors, washed, and then treated with 10 mL of a prewarmed digestion solution containing 0.1% type IV collagenase at 37 °C for approximately 30–40 min. The supernatant was subsequently transferred to a new 50 mL centrifuge tube kept at 4 °C. This digestion process was repeated for the remaining tissue fragments. The resulting supernatant was filtered through 70 μm and 40 μm filters. The cells were seeded in BME2 matrix (R&D Systems, Minneapolis, MN, USA) and cultured in advanced DMEM/F12 medium supplemented with 10 mM nicotinamide (Sigma, Minneapolis, MN, USA), 1.25 mM N-acetyl-L-cysteine (Sigma, Minneapolis, MN, USA), 1:50 B-27 (Gibco, Waltham, MA, USA), 10 nM recombinant human-gastrin I (Peprotech, USA), 100 ngmL^− 1^ recombinant human RSPO1 (MedChemExpress, Monmouth Junction, NJ, USA), 10 µM Y27632 (Sigma, Minneapolis, MN, USA), 1 mM A8301 (Tocris Bioscience, Minneapolis, MN, USA), 50 ngmL^− 1^ recombinant rat epidermal growth factor (EGF; Peprotech, Cranbury, NJ, USA), 50 ngmL^− 1^ recombinant human hepatocyte growth factor (HGF; Peprotech, Cranbury, NJ, USA) and 100 ngmL^− 1^ recombinant human FGF10 (Peprotech, Cranbury, NJ, USA).

### Single-cell suspension preparation

Rat samples were collected and transferred to sterile dishes on ice. Adipose, fibrous, and necrotic tissues were dissected. The remaining tissue samples were cut into small1–2 mm fragments and digested with 1 mL collagenase IV and 100 µL of DNase (Servicebio, China) at 37 °C with oscillation for 15 min. The samples were then passed through a 40 μm sterile strainer, and the cells were removed by centrifugation at 250× g for 5 min. The cells were resuspended in phosphate-buffered saline (PBS), treated with 1 mL ice-cold red blood cell lysis buffer (Servicebio, China), and incubated at 4 °C for 10 min. The resulting single-cell suspension was collected, resuspended in PBS, and subjected to cell counting under a microscope. Trypan blue staining was used to assess cell viability.

### Single-cell RNA sequencing data processing

Single-cell RNA sequencing (scRNA-seq) was conducted by Shanghai Novogene Bioinformatics Co., Ltd. Chromium Single Cell 3’Reagent v3 kits were used for the preparation of barcoded scRNA-seq libraries, according to the manufacturer’s instructions. Cell suspensions were processed using a Chromium Single-Cell Controller (10× Genomics) to generate single-cell gel beads-in-emulsion (GEMs). To ensure the capture of 8,000 cells per library, approximately 12,000 cells were introduced into each channel. After GEM formation, reverse transcription was initiated to produce the barcoded full-length DNA. This was followed by emulsion disruption using a recovery agent and subsequent complementary DNA (cDNA) purification using DynaBeads MyOne Silane Beads (Thermo Fisher Scientific, USA). The cDNA was then subjected to polymerase chain reaction (PCR) amplification and the number of cycles was determined based on the number of recovered cells. The amplified cDNA underwent fragmentation, end repair, adenylation (A-tailing), and ligation to an index adapter, culminating in library amplification. Each library was subsequently sequenced on a HiSeq X Ten platform (Illumina, USA), yielding 150 bp paired-end reads.

### scRNA-seq data analysis

The rat genome (Ensembl v93) was used as the reference genome for our analysis. scRNA-seq data from all samples were normalized using the R package Seurat (version 4.0). For quality control, genes expressed in fewer than three cells, cells with fewer than 500 genes, or cells with more than 25% mitochondrial gene content were excluded from a single sample. Transcript counts for each cell type were normalized to 40,000. Dimensionality reduction was achieved using Principal Component Analysis (PCA). Unsupervised clustering was performed on cells originating from the same major cell type, followed by t-distributed Stochastic Neighbor Embedding (t-SNE), graph-based clustering, and marker gene analysis to identify cell subtypes. Cell clustering was conducted using the FindClusters function, and differential gene expression (DEG) between clusters was identified using the FindAllMarkers function with parameters set at min.pct = 0.25 and logFC.threshold = 0.5. For the statistical analysis, *p*-values were calculated using the nonparametric Wilcoxon rank-sum test.

### Characterization of cell populations

The following cell types were classified on the basis of established markers: T cells (CD3D, CD3E), B cells (CD79A, CD19), natural killer (NK) cells (NKG7, NCR1), neutrophils (CSF3R, S100A9), dendritic cells (CLEC9A, TLR7), bile duct epithelial cells (KRT7, TM4SF1), monocytes (FCGR3A, CD68), HPCs (EPCAM, SOX9, CLDN4), myofibroblasts (BGN, ACTA2, COL6A2), hepatocytes (ALB, HNF4A, HP), plasma cells (MZB1, SSR4), and endothelial cells (ENG, VWF, CDH5).

### Construction of single-cell trajectories

Cell differentiation trajectories were reconstructed using Monocle 3. Genes exhibiting significant variation along the pseudotime trajectory were identified through graph-autocorrelation analysis (*q*-value < 0.01). Dynamic expression patterns of these genes were visualized using plot_genes_in_pseudotime.

### CIBERSORTx analysis

Two datasets were utilized in this study. Dataset 1 was obtained from the Liver Hepatocellular Carcinoma (LIHC) dataset of The Cancer Genome Atlas (TCGA), which was accessed via the UCSC Xena data platform (https://xena.ucsc.edu). Dataset 2 was derived from the GSE27150 dataset of the Gene Expression Omnibus (GEO) database (https://www.ncbi.nlm.nih.gov/geo/).

To evaluate the relative abundance of each cell type identified, raw gene expression data were first normalized via removal of lowly expressed genes, batch effect correction, and log transformation. An appropriate cell type reference matrix was selected. The preprocessed gene expression data and selected reference matrix were input into the CIBERSORTx algorithm for analysis. The output by CIBERSORTx were analyzed, and the relative abundances of cell types in different samples were visualized. Based on the median of relative cell abundance, samples were divided into high-abundance (> 50%) and low-abundance (≤ 50%) groups. Kaplan–Meier survival analysis was conducted to assess the prognostic differences between both groups.

### Immunofluorescence staining

Immunofluorescence staining was performed using the following primary antibodies: rabbit anti-α-smooth muscle actin (α-SMA; 1:500 dilution, Invitrogen, USA) and anti- platelet-derived growth factor receptor alpha (PDGFRA; 1:200 dilution, Invitrogen, USA), as reported previously [30]. Tissues were incubated overnight at 4 °C, followed by incubation with fluorescently labeled secondary antibodies for 1 h at room temperature. Nuclear staining was achieved using DAPI. All slides were visualized using a STELLARIS 5 confocal microscope (Leica Microsystems, Germany).

### Real-time quantitative polymerase chain reaction

Total RNA was extracted from cultured cell lines or tissues using TRIzol reagent (Invitrogen, USA), following the manufacturer’s protocol, and quantified using a NanoDrop 1000 spectrophotometer (Thermo Fisher Scientific, USA). cDNA was synthesized using the Bestar™ qPCR RT kit (DBI Bioscience, Germany). Subsequent reverse transcription PCR (RT-PCR) was performed using a SYBR Green PrimeScript RT-PCR kit (Takara Bio, Japan). The gene expression of β-actin was used as an endogenous control for normalization via the 2^−ΔΔCt^ method.

### Transcriptome sequencing

Total RNA was isolated using TRIzol reagent (Invitrogen, USA) and treated with RNase-free DNase I (Thermo Fisher Scientific, USA) to eliminate genomic DNA contamination. Sequencing libraries were constructed from 1 µg of total RNA using the Illumina TruSeq Stranded mRNA LT kit (Illumina, USA) following the manufacturer’s instructions. Paired-end sequencing (150 bp) was performed using the Illumina NovaSeq 6000 platform. Raw reads were quality-controlled using FastQC (version 0.11.9) and those containing > 5% bases below Q30 were filtered out. Adapter sequences were trimmed using Trimmomatic (version 0.39). Clean reads were aligned to the Ensembl rat reference genome (Rnor_6.0) using STAR aligner (version 2.7.10b) with the parameters set to outFilterMismatchNoverLmax 0.05. Transcript abundance was quantified as transcripts per million (TPM) using Salmon software (version 1.6.0). DEGs were identified using DESeq2 (version 1.34.0), with thresholds of |log_2_ (FC)| >1 and false discovery rate (FDR) < 0.05. Bioinformatic analyses were conducted by Hangzhou LC Biotechnology Co., Ltd.

### Luciferase assay

Luciferase reporter assays for farnesoid X receptor (FXR) activity were conducted according to the following protocol. HEK293T cells were seeded at a density of 1 × 10^4^ cells/well. Transfections were performed using Lipo8000™ reagent following the manufacturer’s instructions. Each well received a mixture containing 100 ng each of FXR, retinoid X receptor, apical sodium-dependent BA transporter, and small heterodimer partner plasmids, along with 2–3 ng of a Renilla luciferase plasmid for co-transfection. After 4 h, the culture medium was replaced and each well was supplemented with 100 µM GCDCA and 20 µM chenodeoxycholic acid. After 24 h, luciferase activity was measured using the Dual-Glo Luciferase Assay System (Yeasen Biotech, China).

### Tango-G protein-coupled receptor assay

HTLA cells, a HEK293 cell line stably expressing β-arrestin-TEV and tTA-luciferase (courtesy of Dr. Yi Rao, Capital Medical University) were transfected with 200 ng of the G protein-coupled receptor-sphingosine-1-phosphate receptor 2 (GPCR-S1PR2) plasmid and 400 ng of polyethyleneimine [31]. After 24 h, the medium was replaced with GCDCA to stimulate the cells. For the luciferase activity assay, the supernatant was removed and 50 µL of Bright-Glo reagent (Promega Corporation, USA) was added to each well. Following a 20-min incubation at room temperature, luminescence was measured using a Centro microplate luminometer. Fold activation was determined by normalizing the relative luminescence units of each experimental condition to those of the control samples containing medium only.

### Colony formation assay

Cells were seeded in culture dishes at a density of 5 × 10^2^ cells/well, treated with supernatant derived from GCDCA conditioned medium, and incubated at 37 °C for 7 days. The cells were subsequently fixed with 4% paraformaldehyde and stained with 0.1% crystal violet. Colonies were counted using ImageJ software.

### Sphere formation assay

Cells were seeded at a density of 1 × 10^3^ cells per well in ultralow attachment six-well plates. The culture medium consisted of serum-free DMEM/F12 enriched with 20 ng/mL recombinant human insulin-like growth factor-1 (IGF-1), 20 ng/mL recombinant human EGF, 4 µg/mL heparin sodium salt, 1% (v/v) MEM non-essential amino acid solution, 0.1 mM β-mercaptoethanol, and 1×GlutaMAX™-I supplement. After cell stabilization, the GCDCA-CM was added. After 7 days, the number of spheroids was quantified using a microscope.

### Measurement of liver BA

Samples were collected from 14 patients with primary liver cancer, and stored at − 80 °C for subsequent BA measurement. Briefly, 10 mg of tissue was weighed and placed in a centrifuge tube with approximately 25 mg of pre-chilled beads and 20 µL of ultrapure water, then homogenized using a bead beater. In the centrifuge tube, 180 µL of a mixed solvent of acetonitrile/methanol (v/v = 8:2) containing 10 µL of internal standard was added and homogenized again. After centrifugation at 13,500 rpm for 20 min at 4 °C, 40 µL of the supernatant was transferred to a 96-well plate. Following lyophilization using a freeze dryer, the dried samples were re-suspended in a mixture of acetonitrile/methanol (80/20) and ultrapure water at a 1:1 ratio, and centrifuged again at 13,500 rpm for 20 min at 4 °C. The supernatant was then transferred to a 96-well plate for subsequent analysis. The injection volume for analysis was 5 µL. Hepatic BA concentrations were quantified using high-performance liquid chromatography coupled with tandem mass spectrometry.

### Sirius red staining

Tissue sections were dewaxed in xylene and rehydrated using graded ethanol solutions and distilled water. The sections were stained with Sirius Red, differentiated with glacial acetic acid, dehydrated using an ascending ethanol series, cleared in xylene, and mounted with neutral resin for microscopic evaluation.

### Masson staining

Following standardized dewaxing and rehydration procedures, the sections were stained with Weigert’s iron hematoxylin and differentiated in 1% acidic alcohol until the nuclear structures displayed optimal blue color. After thoroughly rinsing under running water, the sections were subsequently stained with ponceau-acid fuchsin solution and differentiated in 1% phosphomolybdic acid to remove any unbound dye. Collagen fibers were counterstained with light green, briefly differentiated in 1% acetic acid, dehydrated using a graded ethanol series, cleared in xylene, and permanently mounted on a neutral resin for microscopic examination.

### Western blotting

Cells were collected, washed, and lysed using RIPA buffer. Equal amounts of protein were separated by SDS-PAGE and transferred onto nitrocellulose membranes. The membranes were blocked with 5% skim milk in Tris-buffered saline (TBS) for 1 h at room temperature, followed by incubation overnight at 4 °C with the following rabbit-derived primary antibodies: anti-PDGFRA (1:500, Invitrogen), anti-α-SMA (1:1000, Cell Signaling Technology), and anti-GAPDH (1:5000, BioWorld Technology) as a loading control. After washing three times with TBST (TBS containing 0.1% Tween-20), the membranes were incubated with a goat anti-rabbit IgG secondary antibody (1:5000, BioWorld Technology) for 1 h at room temperature. Protein bands were finally detected using an enhanced chemiluminescence (ECL) system (BioEcho Technology).

### Statistical analysis

All experiments were repeated at least three times. Statistical analyses were performed using R version 4.2.1 and GraphPad Prism 9.0. Data are expressed as mean ± standard deviation (SD). For two-group comparisons, a two-tailed Student’s *t*-test or Mann–Whitney U test was used. Multigroup comparisons were performed using one-way analysis of variance or the Kruskal-Wallis test. Survival curves were generated using the Kaplan–Meier method, and between-group differences were assessed using the log-rank test. Spearman’s rank correlation test was used to evaluate variable associations. Statistical significance was set as *p < 0.05, **p < 0.01, and ***p < 0.001.

## Results

### scRNA-seq reveals the heterogeneity and origins of CAFs during hepatocarcinogenesis in rats

**Fig. 1:**
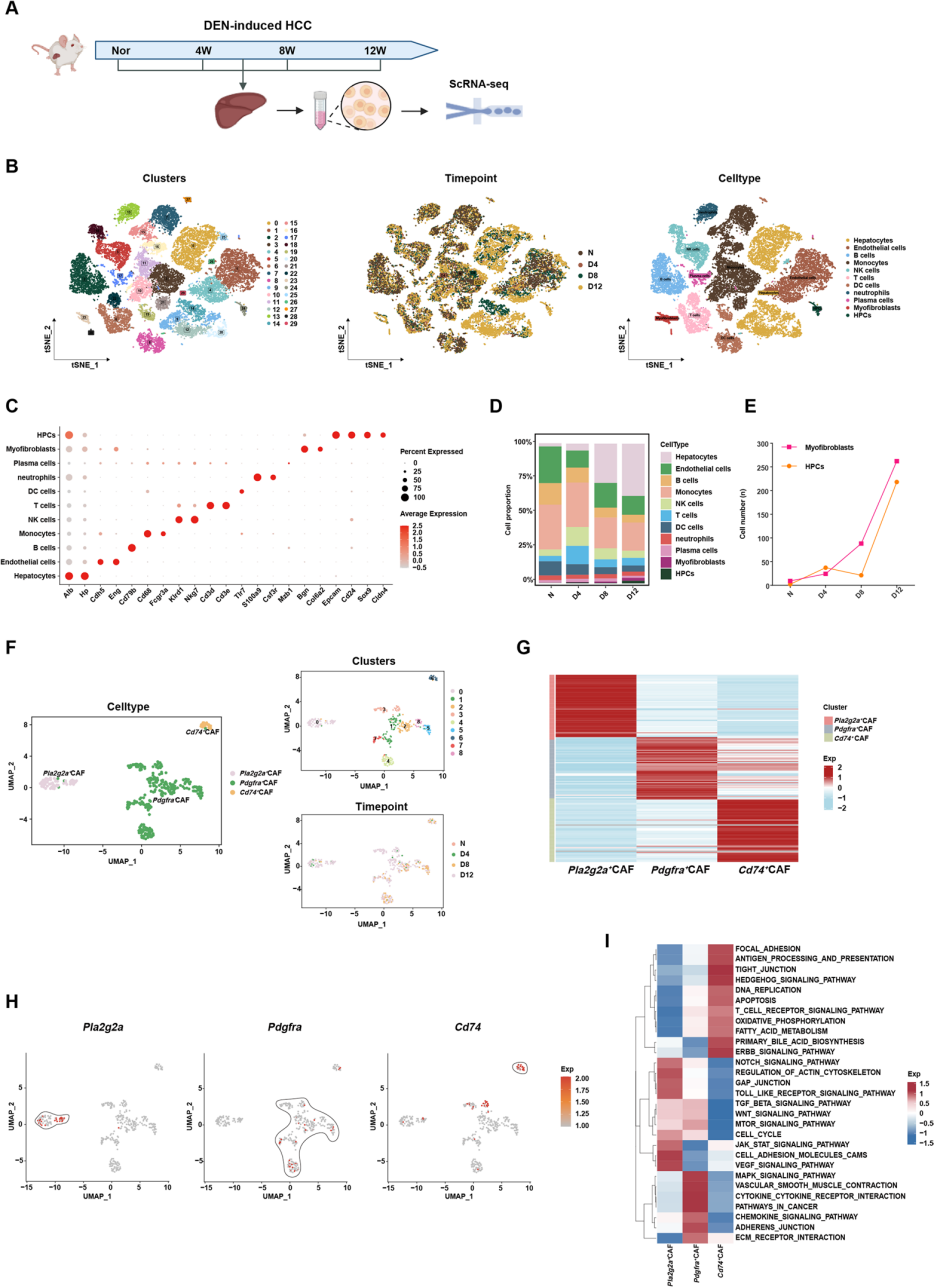
Single-cell RNA sequencing reveals cancer-associated fibroblast (CAF) heterogeneity in a rat model of HCC (**A**) Experimental timeline of scRNA-seq sampling in diethylnitrosamine (DEN)-induced hepatocarcinogenesis in rats. Liver tissues were harvested at weeks 0 (Normal, Nor), 4 (D4), 8 (D8), and 12 (D12) following DEN exposure (**B**) t-distributed stochastic neighbor embedding (t-SNE) plots depicting clustering results for single cells from DEN-treated rats (left panel), the distribution of single cells, colored according to the time points of DEN treatment (middle panel), and cell types from DEN-treated rats, annotated with different colors (right panel) (**C**) Expression of characteristic marker genes in each cell type: HPCs, myofibroblasts, plasma cells, neutrophils, DC cells, T cells, NK cells, monocytes, B cells, endothelial cells, and hepatocytes (**D**) Distribution of the proportions of various cellular subsets at different time points in the DEN-induced rat model (**E**) Dynamic changes in the proportions of HPCs and CAFs in the DEN-induced rat model (**F**) Uniform manifold approximation and projection (UMAP) plots showing the three CAFs, with colors encoding the three clusters (left panel), all fibroblast subpopulations (top right panel), and the distribution of fibroblast subpopulations colored with time points of DEN treatment (lower right panel) (**G**) Heatmap depicting the differentially expressed genes (DEGs) between three CAFs subpopulations (**H**) UMAP plot with color coding representing the expression of marker genes for three subpopulations: *Pla2g2a*, *Pdgfra*, and *Cd74* (**I**) Gene set variation analysis revealing pathway enrichment among different CAFs subpopulations

To investigate the potential mechanisms underlying the role of HPCs in hepatocarcinogenesis, we collected liver tissues at different time points (0, 4, 8, and 12 weeks) from DEN-treated scRNA-seq rats (Fig. 1A). After screening for cell quality and excluding low-quality and doublet cells, 37,930 high-quality cells were retained for subsequent analyses. Subsequently, we performed dimensionality reduction using PCA and clustered the cell populations using the Leiden algorithm, ultimately dividing the cells into 30 subclusters (Fig. 1B). By analyzing the marker genes of each cell sub-cluster, we annotated them as hepatocytes, endothelial cells, B cells, monocytes, NK cells, T cells, dendritic cells, neutrophils, plasma cells, myofibroblasts, and HPCs (Fig. 1C). To reveal the expression characteristics and distribution of different cell types in DEN-treated rat liver tissues, we analyzed the relative abundance of each cell type in the liver cancer microenvironment, reflecting the dynamic changes in cell composition in the liver tissue after DEN treatment (Fig. 1D).

Based on previous studies that reported the trilineage differentiation potential of HPCs derived from chronically damaged livers [10, 32], we focused on two key cell populations in the HCC microenvironment: HPCs and CAFs. Upon dynamically observing changes in the proportions of various cell populations during DEN-induced carcinogenesis in rats, we found that the proportions of HPCs and CAFs showed a time-dependent synchronous upward trend (Fig. 1E). Specifically, with the extension of the DEN induction time, the quantities of these two cell populations increased progressively, indicating a synergistic or regulatory relationship between these cells during HCC development.

Since CAFs, the predominant stromal component in HCC, originate from activated fibroblasts/myofibroblasts during chronic liver injury [24] and critically orchestrate tumor progression through extracellular matrix (ECM) remodeling and paracrine signaling [33], we performed subtyping annotation of fibroblasts to delineate CAFs heterogeneity. Consistent with the previous method, after performing a second PCA dimensionality reduction and clustering using the Leiden method, we divided the fibroblasts into three subclusters (Fig. 1F). By analyzing the marker genes of each subcluster, we annotated them as *Pla2g2a*^+^CAFs, *Pdgfra*^+^CAFs, and *Cd74*^+^CAFs (Fig. 1G, H). Gene set variation analysis (GSVA) was performed to investigate the functions of these subclusters. The subcluster characterized by high expression of *Pla2g2a* had marker genes enriched in pathways related to cell growth, differentiation, and adhesion. The subcluster characterized by high expression of *Pdgfra* had marker genes enriched in pathways related to cell growth, cell differentiation, cell adhesion, angiogenesis, and tumor-related pathways that have been reported to be involved in the TME regulation [34–36]. The subcluster characterized by high expression of *Cd74* had marker genes enriched in pathways related to cell adhesion, antigen presentation, and tight junctions (Fig. 1I). Thus, we hypothesized that during hepatocarcinogenesis in rats, the differentiation trajectory of HPCs may develop towards myofibroblasts.

**Fig. 2:**
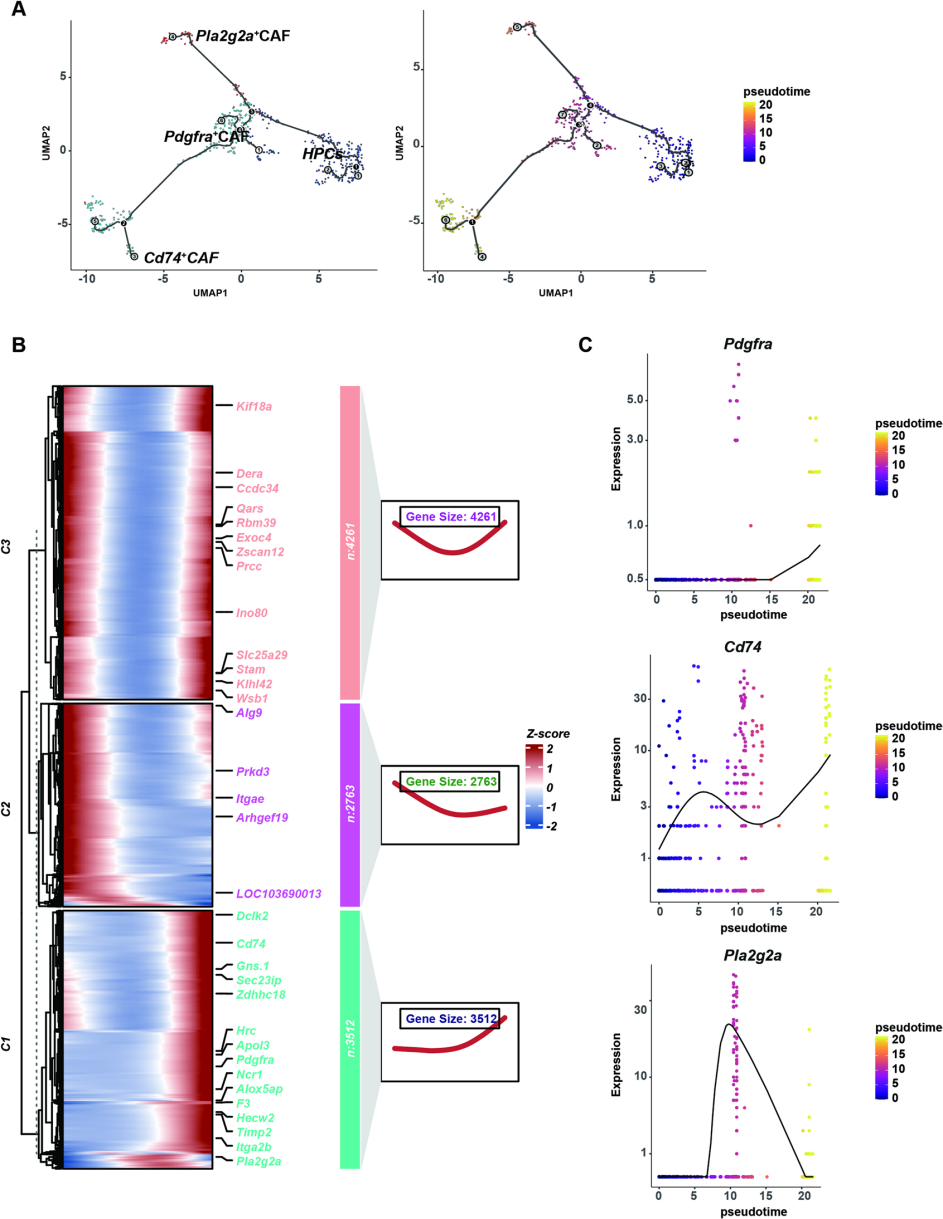
Pseudotime analysis reveals a possible differentiation trajectory from HPCs to CAFs in DEN-induced HCC in rats (**A**) Pseudotime trajectory analysis constructed with Monocle 3, demonstrating the dynamic process of HPC differentiation into three distinct CAF subpopulations. Cells at the end of the branch are represented by dots of different colors (**B**) Pseudotime-ordered heatmap displaying gene expression dynamics across three CAFs subclusters (**C**) Expression of marker genes *Pdgfra*, *Cd74*, and *Pla2g2a* with pseudotime

To further investigate this process, we conducted a trajectory analysis of HPCs and CAFs. Monocle 3 analysis revealed that HPCs may occupy the initial state of the pseudo-time trajectory, whereas *Pdgfra*^+^CAFs, *Pla2g2a*^+^CAFs and *Cd74*^+^CAFs were distributed at the ends of the other two pseudo-time trajectories (Fig. 2A). This distribution pattern suggests that the three CAFs subclusters, *Pdgfra*^+^CAFs, *Pla2g2a*^+^CAFs and *Cd74*^+^CAFs, likely originated from HPCs. The differentiation of HPCs into CAFs may represent a potential pathway for cell fate transformation. Further Monocle 3 analysis delineated the evolutionary trajectory from HPCs to CAFs during liver cancer development and identified three distinct branches, each with unique gene expression patterns (Fig. 2B). To observe the key molecular changes during HPC differentiation, we examined the pseudotime expression trends of three CAFs marker genes. *Pdgfra* expression peaked at the highly differentiated stage, *Pla2g2a* expression peaked during the mid-differentiation phase, while *Cd74* expression peaked at the early differentiation stage (Fig. 2C).

### Identification of HPCs-derived PDGFRA^+^CAF subtypes in human liver cancer via scRNA-seq

We showed the dynamic TME characteristics in a rat liver cancer model, confirming the significant heterogeneity of CAFs in this process. Notably, inflammatory cancer-associated fibroblasts (*Pdgfra*^+^CAFs) originate from the differentiation of HPCs and may be a key subpopulation influencing liver cancer. To verify the conservation of our findings in rats and confirm the relevance of HPCs derived CAFs across different species, we integrated and analyzed single-cell transcriptomic datasets of human liver cancer (GSE125449, GSE136103 and GSE159977). These datasets indicate multiple stages of liver cancer development.

Using a similar method, we first performed PCA reduction and Leiden clustering, eventually dividing the cells into 36 subclusters (Supplementary Fig. S1A). By analyzing the marker genes of each subcluster, we annotated them as cholangiocytes, hepatocytes, myofibroblasts, plasma cells, monocytes, B cells, dendritic cells, neutrophils, HPCs, endothelial cells, NK cells, and T cells (Supplementary Fig. S1B). Consistent with the rat model, human HPCs and CAFs showed simultaneous time-dependent increases as hepatitis progresses to cirrhosis and liver cancer (Supplementary Fig. S1C, D), suggesting that HPCs and CAFs may also have a synergistic or regulatory relationships in the development of liver cancer. After obtaining the fibroblast subclusters, we performed a second PCA reduction and Leiden clustering, annotated based on the marker gene results for each subcluster (Supplementary Fig. S1E). Similar to the rat analysis, the human CAFs could also be divided into three subclusters, including the inflammatory fibroblast subcluster (*PDGFRA*^+^CAFs) observed in rats and the newly emerging subclusters, *RGS5*^+^CAFs and *FGFBP2*^+^CAFs (Supplementary Fig. S1F, G). GSVA revealed that the *PDGFRA*^+^CAFs subcluster was significantly enriched in pathways related to inflammatory responses and growth differentiation (Supplementary Fig. S1H).

Pseudotime analysis of the human dataset using Monocle 3 revealed a possible differentiation trajectory originating from HPCs and point to three CAF subclusters: *PDGFRA*^+^CAFs, *FGFBP2*^+^CAFs, and *RGS5*^+^CAFs (Supplementary Fig. S2A). Further analyses revealed a heterogeneous differentiation trajectory from HPCs to CAFs, highlighting distinct branches that are potentially involved in separate biological processes within the HCC microenvironment, each defined by a unique gene expression profile (Supplementary Fig. S2B). In addition, the expression levels of the three marker genes (*FGFBP2*, *PDGFRA* and *RGS5*) across pseudotime showed a significant trend with the progression of cell differentiation (Supplementary Fig. S2C).

Therefore, we identified an inflammatory cancer-associated fibroblast (iCAF) subcluster (PDGFRA^+^CAFs) that originates from HPCs differentiation in both rat models and human liver cancer, suggesting it is a critical subcluster influencing liver cancer progression.

### Abundant PDGFRA^+^CAFs are associated with poor prognosis in patients with HCC

To investigate the clinical significance of the CAF subtypes, we used CIBERSORTx to evaluate the distribution of various cell types in a LIHC cohort from TCGA and GEO databases. Given the significant heterogeneity of CAFs within the TME, we further divided CAFs into two subtypes: inflammatory CAFs (iCAFs) (characterized by PDGFRA^+^CAFs) and stromal CAFs (characterized by RGS5^+^CAFs). iCAFs have been shown to highly express inflammation-related factors such as IL-6, IL-8, and CXCL12, and primarily participating in inflammatory responses and immune regulation [37, 38]. In contrast, stromal CAFs exhibit increased expression of ECM components, such as POSTN and LUM, promoting tumor cell invasion and metastasis through ECM hardening [39, 40].

**Fig. 3:**
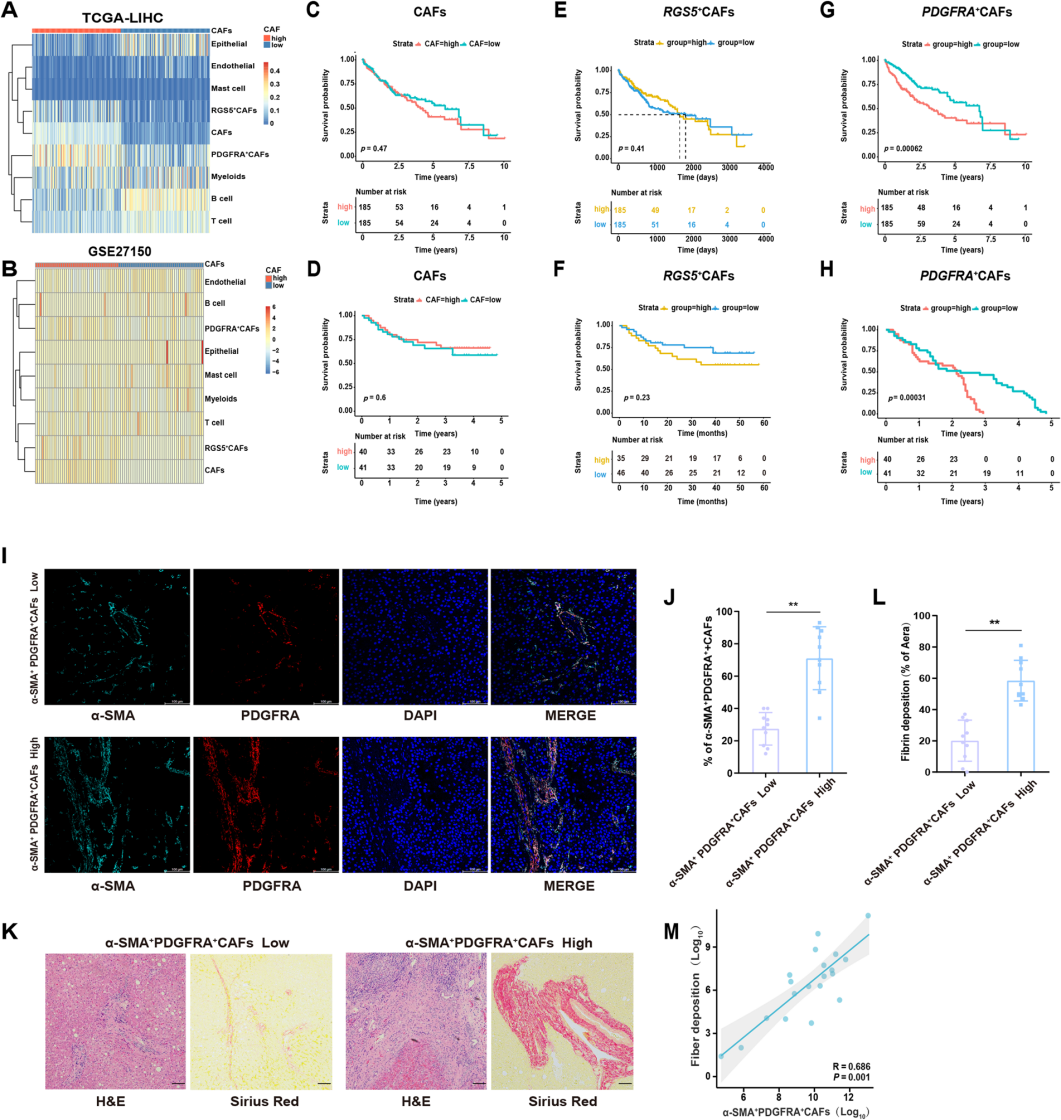
Presence of PDGFRA ^+^α-SMA ^+^CAFs in peritumoral tissue correlates with poor prognosis in patients with HCC (**A**, **B**) Heatmap of cell abundance per sample in the TCGA-LIHC and GEO database cohorts generated by CIBERSORTx, with row z-scores (**C**, **D**) Kaplan-Meier survival plots for patients in the TCGA-LIHC or GEO databases, stratified on the basis of the expression levels of high and low CAF abundance. *P*-values were calculated using the log-rank test (**E**, **F**) Kaplan-Meier survival curves for patients in the TCGA-LIHC or GEO databases, stratified by high and low RGS5^+^expression. *P*-values were calculated using the log-rank test (**G**, **H**) Kaplan-Meier curves for patients in the TCGA-LIHC or GEO databases, grouped by the expression level of PDGFRA^+^CAFs. *P*-values were calculated using the log-rank test (**I**, **J**) Immunofluorescence staining of α-SMA⁺PDGFRA⁺CAFs in peritumoral liver tissues, showing representative images of α-SMA (green) and PDGFRA (red) co-expression. Nuclei were stained with DAPI (blue). Scale bar, 100 μm. Percentage of α-SMA⁺PDGFRA⁺CAFs cells in the two groups. Data are represented as mean ± SD. **p < 0.01 (**K**, **L**) Representative images of H&E staining and Sirius Red staining in different α-SMA^+^ PDGFRA^+^ groups. Percentage area of fibrin deposition in tissues from patients in the low and high groups was analyzed based on Sirius Red staining. Scale bar, 100 μm. Data are represented as mean ± SD. **p < 0.01 (**M**) Correlation analysis between collagen deposition and α-SMA⁺PDGFRA⁺CAF abundance in peritumoral liver tissues using Spearman’s rank correlation coefficient

The TCGA-LIHC cohort mainly included the following cell types: epithelial cells, endothelial cells, mast cells, myeloid cells, B cells, T cells, CAFs, RGS5^+^CAFs, and PDGFRA^+^CAFs (Fig. 3A). Upon dividing the CAFs into high- and low-abundance groups, we found that there were significant differences in cell abundance among the different samples, and the proportion of RGS5^+^CAFs and PDGFRA^+^CAFs in the high-abundance group was significantly higher than that in the low-abundance group. This pattern was also observed in the GSE27150 dataset (Fig. 3B). Upon examining the correlation between the three fibroblast subtypes (total CAFs, iCAFs, and stromal CAFs) and the prognosis of patients with HCC, we found that a higher cumulative expression level of total CAFs was not associated with poorer overall survival (OS) in the TCGA-LIHC cohort (*p* = 0.47) (Fig. 3C). Similarly, high CAFs levels were not significantly correlated with OS in the GSE27150 cohort (*p* = 0.6) (Fig. 3D). Upon evaluating the survival of different CAFs in the cohorts, we found that the median expression stratification of RGS5^+^CAFs showed no correlation with poor OS in the TCGA-LIHC (*p* = 0.41) (Fig. 3E) and GEO cohorts (*p* = 0.23) (Fig. 3F). In contrast, PDGFRA^+^CAFs accumulation was significantly associated with poor OS in the TCGA-LIHC (*p* = 0.00062) and GEO (*p* = 0.00031) cohorts (Fig. 3G, H). Compared with total CAFs and stromal CAFs (RGS5^+^CAFs), iCAF accumulation was significantly associated with poor OS. The higher the proportion of PDGFRA^+^CAFs within the TME, the worse the prognosis, suggesting that PDGFRA^+^CAFs can serve as a predictor of poor prognosis in patients with HCC.

To further verify the potential role of PDGFRA^+^CAFs in the TME of HCC, we performed immunofluorescence staining for α-SMA and PDGFRA to observe the expression of inflammation-associated fibroblasts (Fig. 3I, J). Consistent with our findings in the rat model, we also observed PDGFRA^+^α-SMA^+^ in patients with HCC. After patients were divided into α-SMA^+^PDGFRA^+^low and high groups, H&E and Sirius Red staining were performed. The staining revealed significant differences between the two groups in terms of the pathological features of inflammatory fibroblasts and the distribution of collagen or fibrin deposition in peritumoral tissues. (Fig. 3K, L). Spearman correlation analysis revealed a significant positive association between the infiltration density of inflammatory fibroblasts (α-SMA^+^PDGFRA^+^CAFs) and peritumoral collagen deposition area (*r* = 0.686, *p* = 0.001) (Fig. 3M), suggesting this subset may directly drive hepatic fibrosis via ECM remodeling. These findings indicated that PDGFRA^+^CAFs could serve as potential biomarkers for evaluating fibrosis severity and HCC progression.

### GCDCA promotes the differentiation of HPCs into the PDGFRA^+^CAFs phenotype

**Fig. 4:**
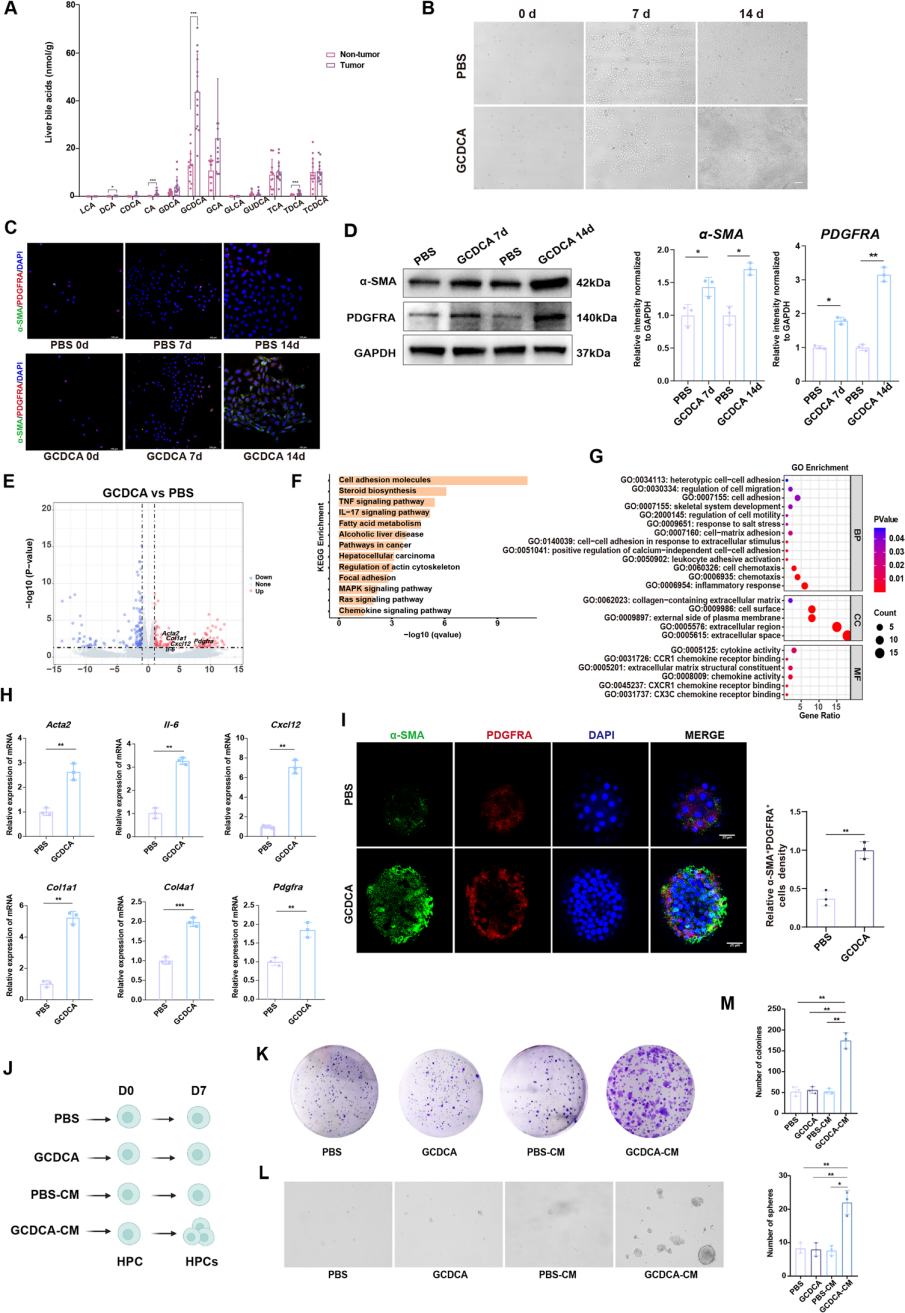
GCDCA induces the differentiation of HPCs into PDGFRA^+^α-SMA^+^CAFs (**A**) Quantification of bile acid levels in liver tissues from patients with primary liver cancer, determined using ultra-performance liquid chromatography-tandem mass spectrometry (n=14). Data are presented as mean ± SD. *p < 0.05, ***p < 0.001 (**B**) Morphological changes in WB-F344 rat HPCs following exposure to 100 µM GCDCA for 7 or 14 days in vitro. Scale bar, 100 μm (**C**) Immunofluorescence staining of α-SMA expression (green) and PDGFRA (red) in WB-F344 cells treated with 100 µM GCDCA for 7 or 14 days in vitro. Scale bar, 100 μm. Nuclei were labeled by DAPI staining (**D**) WB-F344 cells were treated with GCDCA for 7 or 14 days. The protein levels of α-SMA and PDGFRA were assessed by Western blotting. Quantification of image was shown as means ± SD. *p < 0.05, **p < 0.01 (**E**) Transcriptomic sequencing was performed after 14 days of GCDCA treatment. Volcano plot showing differentially expressed genes (DEGs) between WB-F344 cells treated with GCDCA and untreated WB-F344 cells (**F**, **G**) KEGG and GO enrichment analyses were performed on the DEGs among the indicated groups (**H**) The expression of inflammatory fibroblast-associated genes was examined by RT-PCR (*n* = 3). The values are shown as the mean ± SD. **p < 0.01, ***p < 0.001 (**I**) Following an 8-week DEN treatment, primary HPCs were isolated from rat livers and cultured under organoid conditions. These organoids were subsequently exposed to 100 µM GCDCA for two weeks. After treatment, the organoids were subjected to immunofluorescence staining to detect α-SMA (green) and PDGFRA (red). Nuclei were labeled by DAPI staining. Scale bars, 25 μm. Quantification of image was shown as means ± SD. **p < 0.01 (**J**) Experimental design diagram for investigating the effects of different groups (PBS, GCDCA, PBS-CM and GCDCA-CM) on the proliferation of HPCs (**K**, **L**) Colony formation and the sphere-forming ability of different groups (PBS, GCDCA, PBS-CM and GCDCA-CM) cells at 7 day. HPCs were treated with PBS, GCDCA, conditioned medium (CM) from GCDCA-generated CAFs, or PBS-CM from WB-F344 cells, respectively. Scale bar, 100 μm (**M**) Quantification of colony formation and sphere assay (*n* = 3). Data are expressed as mean ± SD. *p < 0.05, **p < 0.01

GCDCA has been shown to significantly elevated levels reported in patients with cirrhosis and HCC [24, 35, 41]. After analyzing liver tissue samples from 14 patients with primary liver cancer, we found that GCDCA concentration in human HCC tissues reached approximately 70 nmol/g (equivalent to ~ 70 µM), whereas it was only about 1–10 nmol/g (equivalent to ~ 1–5 µM) in adjacent non-tumor tissues (Fig. 4A). Consistently, it was reported that GCDCA levels in patients with cholestatic chronic liver disease or HCC are commonly reported to be range 10–200 µM [24, 41, 42]. It suggested that there was a significant GCDCA accumulation during HCC progression.

It was reported that HPCs can abnormally differentiate into myofibroblasts in chronic liver disease, responding to microenvironmental signals [10].In our study, the trajectory analysis of HPCs suggested a potential differentiation to CAFs. Therefore, we hypothesize that GCDCA can promote HPCs differentiate into CAFs in the tumor environment. Accordingly, we conducted an in vitro differentiation assay to evaluate the effect of GCDCA on the HPC, using WB-F344 cells (rat liver progenitor cell line). We treated the cells with different concentrations of GCDCA (1 µM, 10 µM, 100 µM) (Fig. S3A). We found that the cells gradually exhibited characteristic myofibroblastic morphological features on the 14th day after 100 µM GCDCA treatment. The WB-F344 cells had a typical polygonal shape under normal conditions. However, after GCDCA treatment, cell morphology gradually changed to a myofibroblast appearance (Fig. 4B), suggesting that GCDCA may induce the differentiation of HPCs into myofibroblasts To validate this phenotypic change, we performed immunofluorescence staining for α-SMA, a marker molecule of myofibroblasts [43]. Given the significant role of PDGFRA^+^ CAFs in assessing the degree of fibrosis and the progression of HCC in our findings, we also examined the co-expression of α-SMA and PDGFRA. The results revealed that in the GCDCA group, both expression of α-SMA and PDGFRA the intensity of fluorescent signals were significantly increased (Fig. 4C), providing preliminary evidence that GCDCA could promote the differentiation of HPCs into CAFs. To further investigate the effect of GCDCA on HPCs differentiation, we performed Western blot analysis (Fig. 4D). The results confirmed that the GCDCA-induced differentiated phenotype became more pronounced over time, as evidenced by upregulation of α-SMA and PDGFRA protein levels.

Since iCAFs are characterized by high expression of α-SMA, pro-inflammatory factors such as IL-6 and CXCL12 [42], and ECM components such as collagen COL1A1 and COL4A1 [35, 36], we further investigated the changes in these gene expression in transcriptomic analysis. Genes related to the inflammatory fibroblast phenotype, such as *Acta2*, *Col1a1*, *Cxcl12*, *Il-6*, and *Pdgfra* were significantly upregulated upon stimulation with GCDCA (Fig. 4E). KEGG and GO enrichment analyses to observe the changes in signaling pathways post-treatment. The pathways upregulated by GCDCA were mainly enriched in cell adhesion, inflammation, proliferation, and tumor-related pathways (Fig. 4F, G). Next, we detected the expression levels of inflammatory fibroblast-related genes by RT-PCR. The genes of inflammatory fibroblasts (including *Acta2*, *Il-6*, *Cxcl12*, *Col1a1*, and *Pdgfra*) were significantly up-regulated under GCDCA (Fig. 4H). Next, we used an organoid model derived from primary HPCs isolated from rats subjected to 8 weeks of DEN induction (Fig. S3B). These organoids were treated with GCDCA for 14 days. Immunofluorescence staining revealed enhanced co-expression of α-SMA and PDGFRA in the GCDCA-treated group, along with an increase in organoid diameter, suggesting a pro-inflammatory and potentially pro-proliferative role (Fig. 4I). To determine whether this pro-proliferative effect depends on CAFs paracrine signaling, we designed four groups: PBS, GCDCA, PBS-conditioned medium (PBS-CM), and GCDCA-conditioned medium (GCDCA-CM) (Fig. 4J). Colony formation and spheroid formation assays demonstrated that GCDCA-CM significantly promoted HPC proliferation, whereas GCDCA alone showed minimal effect (Fig. 4K-M). These results indicate that GCDCA primarily exerts a pro-differentiation effect, while its pro-proliferative role is likely mediated by paracrine factors secreted by differentiated CAFs. This further showed the critical role of GCDCA in driving HPCs differentiation into inflammatory CAFs.

### GCDCA promotes liver fibrosis and hepatocarcinogenesis in a rat model of primary liver cancer

**Fig. 5:**
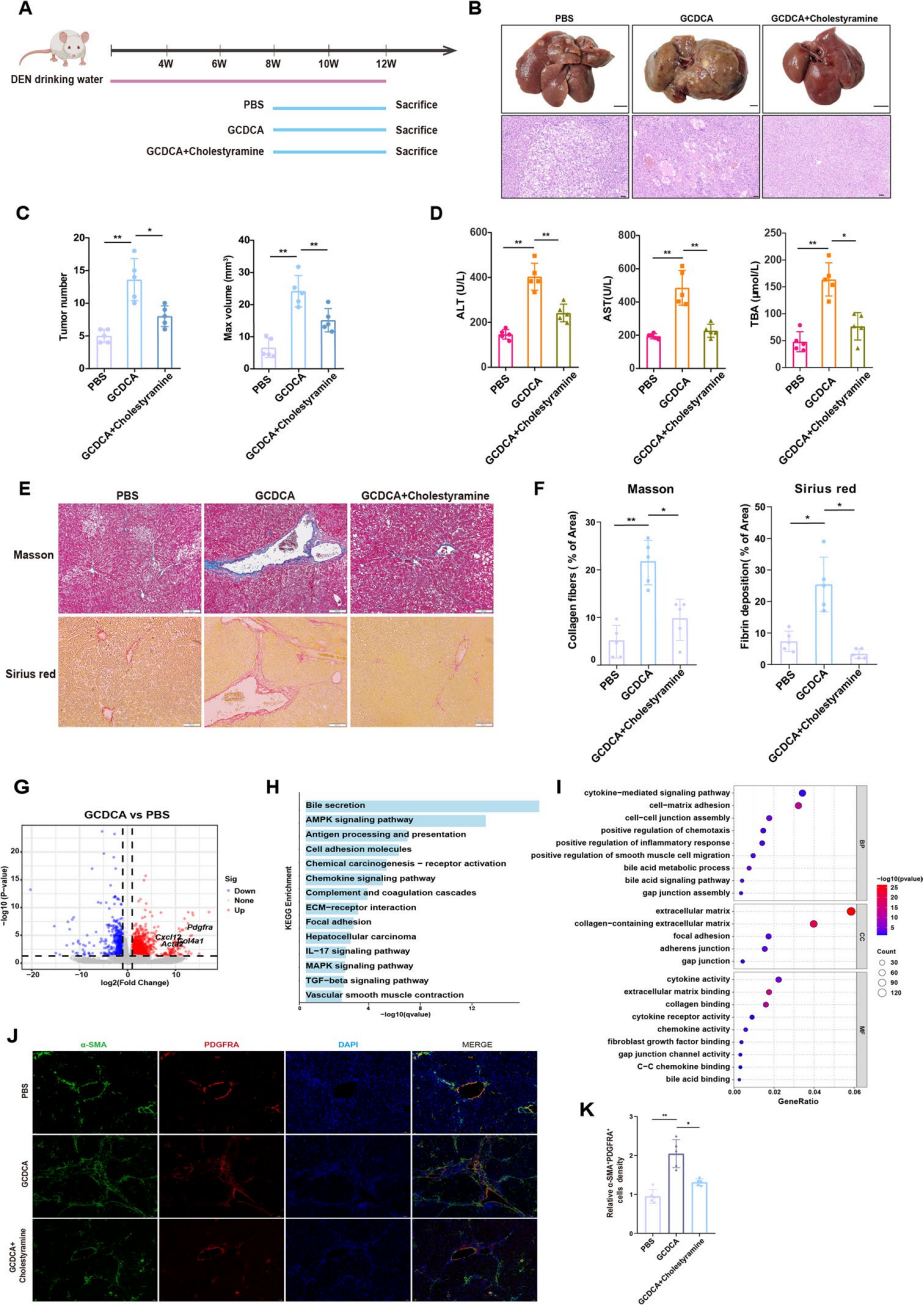
GCDCA promotes hepatocarcinogenesis in a DEN-induced primary liver cancer model (**A**) Schematic representation of the GCDCA intervention in a DEN-induced hepatocarcinogenesis rat model. GCDCA (8 µmol/100 g, i.p.) was administered via intraperitoneal injection continuously for 4 weeks from week 8 to week 12. Co-treatment with a 1.2% cholestyramine diet for 4 weeks (**B**) Gross liver morphology and H&E staining of liver tissue in the different groups at week 12. Scale bars, 1 cm and 50 μm, respectively (**C**) Statistical analysis of the number of nodules and maximum volume in the different groups (*n* = 5 per group). Data are shown as the mean ± SD. *p < 0.05, **p < 0.01 (**D**) Serum alanine transaminase (ALT), aspartate transaminase (AST), and total bile acid (TBA) levels with and without cholestyramine treatment. Data are shown as the mean ± SD. *p < 0.05, **p < 0.01 (**E**) Masson’s trichrome and Sirius Red staining of hepatic collagen deposition with or without cholestyramine treatment. Scale bar, 200 μm (**F**) Percentage area of collagen and fibrin deposition was analyzed. Data are represented as mean ± SD. *p < 0.05, **p < 0.01 (**G**) Volcano plot shows the comparison of DEGs between the liver tissues of rats treated with GCDCA for 4 weeks and the untreated liver tissues (**H**, **I**) Functional enrichment analysis of differentially DEGs (**J**) Immunofluorescence analysis of inflammatory fibroblasts (α-SMA^+^PDGFRA^+^) with or without cholestyramine treatment. Nuclei were counterstained with DAPI (blue). Scale bar, 50 μm (**K**) Percentage of inflammatory fibroblasts (α-SMA^+^PDGFRA^+^) was analyzed and quantification of image was shown as means ± SD. *p < 0.05, **p < 0.01

To further investigate the role of GCDCA in hepatocarcinogenesis, we employed a DEN-induced primary liver cancer rat model and the GCDCA intervention model was established (Fig. 5A). To determine whether GCDCA is directly involved in tumorigenesis, the experimental group was supplemented with cholestyramine, is a BA-binding resin that can inhibit intestinal BA absorption, leading to increased fecal BA excretion [44]. At 12 weeks, macroscopic analyses showed that GCDCA treatment significantly increased tumor incidence, with the maximum tumor volume reaching 20 mm³. Histopathological examination of the DEN-induced tumors in the control group revealed scattered tumor cells forming small solid clusters, whereas the tumor cell density in the GCDCA-treated group significantly increased with focal necrosis and bleeding (Fig. 5B, C). In contrast, the cholestyramine combination group showed significantly reduced tumor incidence and maximum volume. Microscopic evaluation further showed diminished tumor cell degeneration and necrosis, with a significant decrease in nodule count in the cholestyramine-treated group (Fig. 5B). To assess the severity of hepatic injury, serum ALT and AST levels were measured. GCDCA treatment exacerbated DEN-induced hepatotoxicity and significantly elevated ALT, AST, and TBA levels, whereas cholestyramine combination therapy effectively attenuated this damage (Fig. 5D). These findings suggested that GCDCA accelerates hepatocarcinogenesis by promoting BA accumulation and aggravating hepatic injury, whereas cholestyramine protects against enterohepatic BA recirculation.

Masson’s trichrome and Sirius Red staining were performed to evaluate fibrotic remodeling during hepatocarcinogenesis. Compared to controls, GCDCA-treated livers showed increased fibrosis, characterized by increased fibrous septal formation. In contrast, cholestyramine supplementation significantly reduced collagen deposition, indicating reduced fibrosis (Fig. 5E, F).

Furthermore, we performed transcriptomic analysis on rat GCDCA-treated and PBS groups to observe the differentiation process of HPCs into CAFs. Volcano plot results revealed that GCDCA treatment promoted the expression of fibrotic and inflammatory markers such as *Acta2*, *Pdgfra*, *Col4a1* and *Cxcl12* (Fig. 5G). Subsequent KEGG and GO enrichment analyses demonstrated that the pathways upregulated by GCDCA were primarily associated with in bile acid secretion, antigen presentation, cell adhesion, inflammatory response, cellular proliferation, and oncogenic signaling (Fig. 5H, I). Consistent with our previous observations in rat cell line. Immunofluorescence co-staining for α-SMA and PDGFRA showed that GCDCA group significantly increased the expression of α-SMA^+^ PDGFRA^+^ inflammatory fibroblasts, while cholestyramine decreased their expression (Fig. 5J, K). These results indicated that GCDCA promoted the phenotypic transformation of inflammatory fibroblasts in vivo.

### GCDCA induces the differentiation of HPCs into PDGFRA^+^CAFs by activating the S1PR2 receptor

**Fig. 6:**
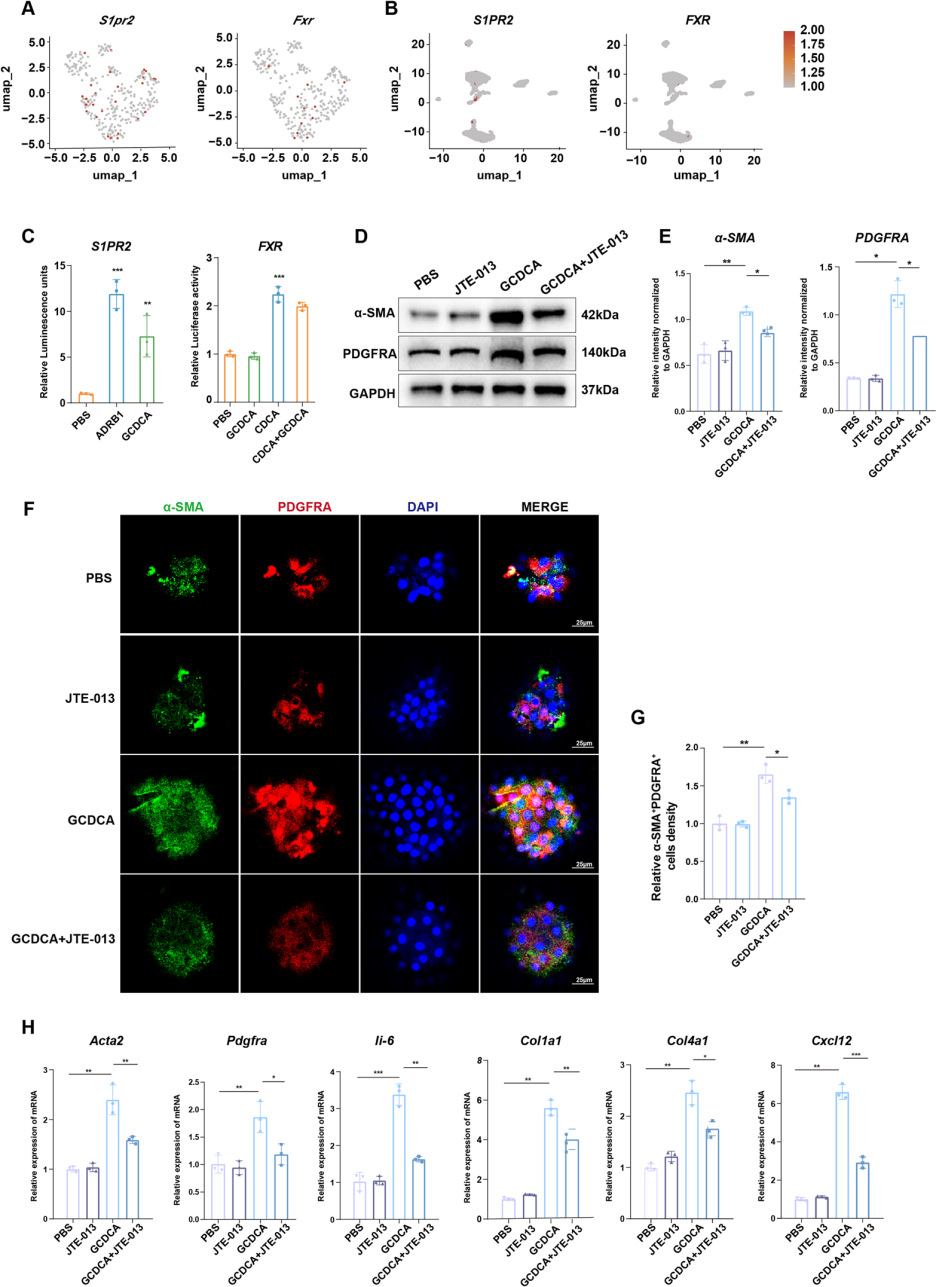
GCDCA induces the differentiation of HPCs into PDGFRA^+^α-SMA^+^CAFs by activating the S1PR2 receptor (**A**, **B**) UMAP plot with color coding representing the expression of bile acid receptors FXR and S1PR2 in rat (**A**) and human (**B**) HPCs (**C**) Tango GPCR assay showing S1PR2 activation in HTLA cells transfected with the S1PR2-Tango plasmid (left). Norepinephrine-treated cells served as positive controls (*n* = 3). Luciferase reporter assay assessing FXR transcriptional activity in cells treated with chenodeoxycholic acid agonist (20 µmol/L) and GCDCA (100 µmol/L) stimulation (right) (*n* = 3). Data are shown as the mean ± SD. **p < 0.01, ***p < 0.001 (**D**, **E**) WB-F344 cells were treated with GCDCA for 14 days. GCDCA groups were pretreated with 10 µM JTE-013 for 1 h. Protein levels of α-SMA and PDGFRA were evaluated by Western blotting. Quantification of image was shown as means ± SD. *p < 0.05, **p < 0.01 (**F**, **G**) Primary HPCs-organoid isolated from rats treated with DEN for 8 weeks were pre-treated with the inhibitor JTE-013 for 1 h prior to GCDCA stimulation, followed by 14 days of culture. Co-expression of α-SMA (green) and PDGFRA (red) was assessed by immunofluorescence staining. Scale bars, 25 μm. Quantification of image was shown as means ± SD. *p < 0.05, **p < 0.01 (**H**) RT-PCR analysis of inflammatory fibroblast marker expression after GCDCA treatment, with or without 1 h pretreatment with 10 µM JTE-013 (*n* = 3). Data are shown as the mean ± SD. *p < 0.05, **p < 0.01, ***p < 0.001

BAs crucial signaling molecules that participate in various metabolic processes and maintain homeostasis by activating specific receptors [45, 46]. BA receptors are divided into two main categories: membrane (G protein-coupled receptor 5 and S1PR2 and nuclear (FXR, pregnane X receptor, and constitutive androstane receptor) receptors [47]. To investigate which receptor mediates GCDCA-induced differentiation of HPCs into inflammatory fibroblasts, we first analyzed BA receptor expression in HPCs using rat liver and human scRNA-seq. The results showed that S1PR2 and FXR receptors were expressed in both rats and humans HPCs (Fig. 6A, B). Subsequently, we established FXR and S1PR2 detection systems in HEK293T and HTLA cell lines. Upon evaluating the transcriptional activity of FXR using the luciferase reporter system in HEK293T cells, we found that GCDCA stimulation had no significant effect on FXR luciferase activity (Fig. 6C). Tango-GPCR analysis showe that HTLA cells transfected with the S1PR2-Tango plasmid showed significantly enhanced fluorescence intensity after GCDCA stimulation (Fig. 6C). These results suggested that GCDCA exerts its biological effects by activating the S1PR2 receptor rather than the FXR receptor, consistent with those of previous studies demonstrating the biological effects of S1PR2-mediated GCDCA in intestinal organoid models [48].

JTE-013, a specific S1PR2 antagonist, has been shown to impact cellular processes such as proliferation, migration, and inflammatory responses [49, 50]. To investigate the role of S1PR2 in the GCDCA-mediated induction of a CAF-like phenotype, we first performed Western blot analysis. The results showed that the S1PR2 inhibitor JTE-013 markedly attenuated the GCDCA-induced pro-fibroinflammatory phenotype, as evidenced by reduced protein levels of α-SMA and PDGFRA (Fig. 6D, E). To further validate the impact of S1PR2 inhibition on HPC differentiation, we employed an in vivo rat model using organoids derived from primary HPCs. Immunofluorescence staining revealed that JTE-013 treatment suppressed the GCDCA-induced differentiation toward an inflammatory phenotype, characterized by decreased co-expression of α-SMA and PDGFRA, compared to the GCDCA group (Fig. 6F, G). Furthermore, RT-PCR analysis demonstrated that GCDCA treatment significantly upregulated the expression of pro-fibroinflammatory markers (*Acta2*, *Pdgfra*, *Ii-6*, *Col1a1*, *Col4a1*, and *Cxcl12*), an effect that was reversed by JTE-013 (Fig. 6H). Collectively, these findings indicate that GCDCA promotes a phenotypic shift toward pro-inflammatory fibroblasts in HPCs by activating S1PR2.

### JTE-013 attenuates GCDCA-induced hepatocarcinogenesis in rats

**Fig. 7:**
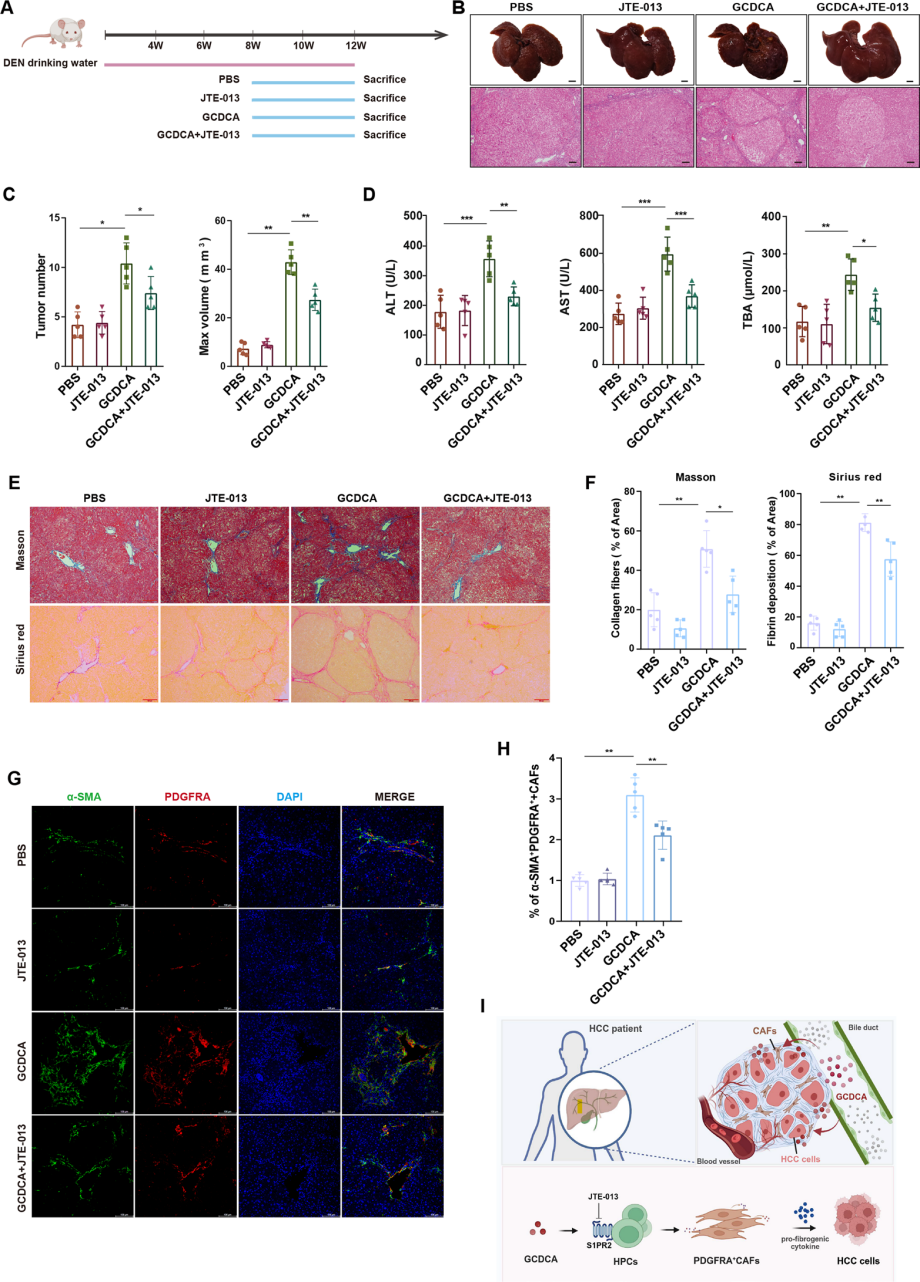
JTE-013 disrupts GCDCA-driven hepatocarcinogenesis via S1PR2 inhibition in rats (**A**) Experimental design of the DEN-induced liver cancer model. GCDCA was administered at week 8, with or without JTE-013 co-treatment (2 mg/kg, i.p., administered 1 h prior to GCDCA (**B**) Macroscopic observation at 12 weeks. Scale bar, 1 cm. H&E staining of liver tumor formation in the different groups. Scale bar, 100 μm (**C**) Hepatic nodule counts and the maximum volume across various groups in a rat model at 12 weeks (*n* = 5 per group). Data are shown as the mean ± SD. *p < 0.05, **p < 0.01 (**D**) Serum AST, ALT, and TBA levels (*n* = 5 per group). Data are shown as the mean ± SD. *p < 0.05, **p < 0.01, ***p < 0.001 (**E**) Representative Masson’s trichome and Sirius Red staining of liver sections at week 12, with or without JTE-013 treatment. Scale bar, 200 μm (**F**) Percentage area of collagen and fibrin deposition was analyzed. Data are represented as mean ± SD.*p < 0.05, **p < 0.01 (**G**) Immunofluorescence analysis of inflammatory fibroblasts (α-SMA^+^PDGFRA^+^) at week 12, with or without JTE-013 treatment. Nuclei stained with DAPI (blue). Scale bar, 100 μm (**H**) Percentage of inflammatory fibroblasts (α-SMA^+^PDGFRA^+^) was analyzed and quantification of image was shown as means ± SD. **p < 0.01 (**I**) Schematic diagram illustrating the process by which GCDCA induces the differentiation of HPCs into myofibroblasts. GCDCA induced the differentiation of HPCs into myofibroblasts, driving their proliferation and malignant transformation through a pro-inflammatory and pro-fibrotic mechanism. The underlying process involves GCDCA-triggered aberrant differentiation of HPCs into PDGFRA^+^CAFs via the bile acid receptor S1PR2, thereby promoting hepatocellular carcinogenesis. Crucially, the accumulation of these PDGFRA^+^CAFs is significantly correlated with poorer patient prognosis and the S1PR2 inhibitor JTE-013 suppresses liver cancer development by alleviating fibrosis and inhibiting this pathogenic phenotypic switch

Considering that the S1PR2 signaling pathway may be involved in the differentiation of HPCs induced by GCDCA and its transition to PDGFRA^+^CAFs, we further investigated its role in hepatocarcinogenesis in DEN-induced rats using the S1PR2-specific inhibitor JTE-013. At 8 weeks, GCDCA intervention was initiated, and each group received GCDCA alone or GCDCA combined with JTE-013. The experiment was continued until week 12 to observe the tumorigenesis in each group (Fig. 7A).

Histopathological analysis with H&E staining showed that the JTE-013 combination group had a significantly reduced number of tumors, improved cell atypia, and a more organized cell arrangement compared to GCDCA alone. JTE-013 treatment significantly inhibited GCDCA-induced liver tumorigenesis (Fig. 7B). Gross observations showed that the incidence of tumors and the maximum tumor volume decreased (Fig. 7B, C). Serum biochemical analysis showed that AST, ALT, and TBA levels in the combination group were significantly lower than those in the GCDCA group, indicating that JTE-013 not only inhibited GCDCA-driven tumorigenesis but also alleviated liver injury (Fig. 7D).

To further investigate the role of S1PR2 in regulating the phenotype of inflammatory fibroblasts in HCC in rats, we evaluated the effects of JTE-013 on hepatic and inflammatory fibroblast phenotypes. Masson’s trichrome and Sirius red staining revealed that JTE-013 treatment significantly alleviated GCDCA-induced liver fibrosis. Microscopic analysis showed that the fibrotic septum was reduced, lobular structure was restored, and collagen fiber deposition was significantly reduced (Fig. 7E, F). Immunofluorescence staining to detect the expression of inflammatory fibroblasts in rat liver tissue showed that the number of α-SMA^+^PDGFRA^+^ cells in the JTE-013 treatment group was significantly reduced compared with that in the GCDCA alone (Fig. 7G, H), indicating that JTE-013 effectively inhibited GCDCA-induced inflammatory fibroblast differentiation.

In summary, we found that GCDCA can induce abnormal differentiation of HPCs into PDGFRA^+^CAFs in the cholestasis microenvironment, thus promoting the hepatocarcinogenesis (Fig. 7I). In addition, this study also elucidates the key regulatory role of the GCDCA-S1PR2 signaling axis in the hepatocarcinogenesis, providing a potential intervention target for the treatment of HCC.

## Discussion

HPCs exhibit multilineage differentiation potential during chronic liver injury. Under physiological conditions, HPCs contribute to tissue repair by differentiating into hepatocytes or bile duct epithelial cells [7, 9]. However, when differentiation is blocked, HPCs may transform into malignant tumor-initiating cells [11, 12]. Recent studies have shown that HPCs can differentiate into α-SMA^+^ myofibroblasts and participate in TME formation [10], enhancing our understanding of the role of HPCs in both tissue repair and pathological transformation.

In this study, we investigated the mechanism of action by which HPCs contribute to CAFs differentiation and their role in the HCC microenvironment using scRNA-seq. Dynamic observations of DEN-induced liver cancer revealed a simultaneous time-dependent increase in the number of HPCs and CAFs, suggesting a possible synergistic effect during HCC. Heterogeneity analysis of CAFs subpopulations in the human dataset identified three subtypes: the *Pdgfra*^+^CAFs subgroup in rats and two newly identified human subtypes, *RGS5*^+^CAFs and *FGFBP2*^+^CAFs. Pseudotime trajectory analysis suggested that the *Pdgfra*^+^CAFs subgroup may have originated from HPCs, a differentiation pattern that was also conserved in humans. Thus, we infered iCAFs (*PDGFRA*^+^CAFs) possible derived from HPCs differentiation in the HCC microenvironment that may represent a key subgroup influencing HCC.

CAFs are typically abnormally activated cells capable of producing large amounts of ECM and exhibit myofibroblastic characteristics [16, 51, 52]. Recent studies have highlighted the phenotypic and functional heterogeneity of CAFs. For example, scRNA-seq analysis of liver metastasis identified three CAF subpopulations: myofibroblasts, iCAFs, and portal fibroblasts. Among these, iCAFs have been shown to promote tumor progression through direct HGF secretion, mediating CAF-tumor cell interactions [53]. In this study, we examined HPCs-derived CAFs subpopulations at different time points in a DEN-induced primary rat model of HCC, which revealed significant phenotypic heterogeneity. Notably, we identified unique iCAFs characterized by PDGFRA expression. These iCAFs express proinflammatory genes and modulate the inflammatory microenvironment in fibrotic livers, thereby promoting tumorigenesis. PDGFRA is a transmembrane tyrosine kinase receptor that activates the PI3K/Akt, RAS/MAPK, and JAK/STAT signaling pathways to promote cell proliferation and survival [54]. Several studies have provided functional and molecular evidence supporting iCAF-mediated tumorigenesis. In breast cancer and pancreatic ductal adenocarcinoma, iCAFs influence the immunosuppressive microenvironment by secreting inflammatory factors such as IL-6 [37, 38]. In colorectal cancer, iCAFs enhance chemotherapeutic resistance by producing IL-6 and CXCL12 [227]. Mechanistically, this iCAFs phenotype is dependent on IL-1β-induced NF-κB activation, with down-regulation of IL-1 receptors in pancreatic stellate cells and colon fibroblasts resulting in reduced inflammatory potential and myeloid infiltration [55, 56]. These findings underscore the therapeutic relevance of iCAFs in tumorigenesis.

We further investigated the underlying mechanisms by which abnormal differentiation of HPCs contributes to the development of HCC in the context of cholestasis. It was found that GCDCA may promote HCC by inducing the differentiation of HPCs into iCAFs. Morphological and immunofluorescence analyses revealed that GCDCA induced the morphological transformation of HPCs into myofibroblast-like cells. Subsequent sphere and colony formation assays suggested that GCDCA promotes the proliferation and stemness of HPCs by inducing CAFs-mediated secretion. Furthermore, transcriptomic sequencing showed significant upregulation of inflammatory fibroblast-related genes in response to GCDCA treatment. Enrichment analysis revealed that the GCDCA activation pathway was mainly related to cell adhesion, inflammation, and tumorigenesis. In rats, GCDCA significantly promoted hepatocarcinogenesis and the transformation of inflammatory phenotypes (α-SMA^+^PDGFRA^+^CAF). These findings confirm that HPCs-induced CAFs play an important role in the progression of fibrosis to liver cancer in a cholestatic environment.

Currently, research on bile acid mediated regulation of the HCC microenvironment primarily focuses on immune responses, particularly the regulation of T cell functions. Bile acids may promote HCC progression by suppressing the activity of tumor-specific T cells [41]. Results from Fig. S3A-B indicate that GCDCA may also function as an immune regulator in the tumor microenvironment. GCDCA not only acts on hepatocytes and fibroblasts but also upregulates transcripts associated with multiple immune cell populations. These findings suggest that GCDCA may promote hepatocarcinogenesis by coordinating a synergistic interaction among HPCs, fibroblasts, and immune cells within the inflammatory tumor microenvironment.

Furthermore, based on previously published studies, we speculated that the increase in GCDCA levels might be related to changes in the activity of BA-carboxylic acid: amino acid N-acyltransferase (BAAT). As a key enzyme in BA metabolism, BAAT catalyzes the conjugation of BAs with amino acids, such as taurine or glycine. The formation of conjugated BAs significantly enhances the water solubility of BAs and facilitates their transport and biliary excretion [57]. In various liver diseases, including HCC, BA metabolism is often disrupted, leading to the abnormal accumulation of certain BAs, particularly conjugated forms [41]. Previous studies have indicated that BAAT contributes to hepatic immune evasion by elevating the levels of primary conjugated BAs and certain secondary BAs, thereby inhibiting the infiltration and function of tumor-specific T-cells [40]. Therefore, further investigation into whether the abnormal accumulation of GCDCA is regulated by upstream BAAT activity is warranted.

S1PR2 is a GPCR that plays a crucial role in cell signaling by regulating cell migration, proliferation, differentiation, survival, and angiogenesis [58]. Studies have shown that S1PR2 is the primary membrane receptor activated by conjugated BAs in hepatocytes [59]. In rodent primary hepatocytes, conjugated BAs activate S1PR2, modulating downstream ERK1/2 and AKT signaling pathways to regulate liver inflammation under cholestatic conditions [60]. Additionally, the invasive growth of human cholangiocarcinoma cells induced by taurocholic acid is associated with S1PR2-mediated upregulation of cyclooxygenase-2 expression and prostaglandin E2 production [59]. Through scRNA-seq, luciferase reporter assays, and Tango-GPCR experiments, we found that GCDCA primarily drives the differentiation of HPCs into iCAFs via S1PR2 activation. Further investigation revealed that the GCDCA-S1PR2 signaling axis plays a pivotal role in hepatocarcinogenesis. GCDCA regulates the differentiationof HPCs through the S1PR2 receptor and drives their phenotypic transition to iCAFs. These findings elucidate the critical role of the GCDCA-S1PR2 axis in the proliferation and phenotypic plasticity of HPCs.

## Conclusions

We investigated the mechanisms underlying hepatocarcinogenesis in a cholestatic microenvironment. Our findings demonstrated that GCDCA drives the aberrant differentiation of HPCs into PDGFRA⁺CAFs, thereby promoting the initiation and progression of HCC. Furthermore, we revealed the critical regulatory role of the GCDCA-S1PR2 signaling axis in cholestasis-associated hepatocarcinogenesis, providing novel mechanistic insights and identifying potential therapeutic targets for HCC treatment.

## Electronic supplementary material

Below is the link to the electronic supplementary material.

Supplementary Material 1

## Data Availability

The single-cell RNA sequencing data generated in this study have been deposited in the Gene Expression Omnibus (GEO) under accession number GSE218561 (https://www.ncbi.nlm.nih.gov/geo/). All other supporting data are included in the manuscript and its supplementary materials.
