# Supplementary material for: Glycochenodeoxycholic acid promotes hepatocarcinogenesis by inducing hepatic progenitor cell differentiation into cancer-associated fibroblasts via sphingosine-1-phosphate receptor 2 signalling
Source: Biomark Res. 2025 Dec 17;13:157. doi: 10.1186/s40364-025-00873-0 (PMC12709717; doi:10.1186/s40364-025-00873-0)
Supplement: Supplementary file 1 — Supplementary Material 1 [file 40364_2025_873_MOESM1_ESM.docx]

**Supplementary Fig. 1. scRNA-seq analysis of CAF heterogeneity during human hepatocarcinogenesis**

(A) scRNA-seq analysis across key pathological stages in the development and progression of liver cancer, including normal liver, hepatitis, and cirrhosis. (A) t-SNE plots displaying the clustering results of single cells from the human liver cancer database, colored with clusters (left panel), the distribution of single cells, colored with points from different patients (middle panel), and displaying the major cell types (right panel).

(B) Expression levels of cell markers in various cell types, annotated as cholangiocytes, hepatocytes, myofibroblasts, plasma cells, monocytes, B cells, dendritic cells, neutrophils, hepatic progenitor cells, endothelial cells, NK cells, and T cells, respectively.

(C) Relative proportional distribution of major cell types during hepatocarcinogenesis.

(D) Dynamic proportion changes of HPCs and CAFs during hepatocarcinogenesis.

(E) UMAP plot of fibroblasts, with colors showing three major clusters (left panel), all subpopulations of fibroblasts (top right panel), and the distribution of fibroblast subpopulations, colored with different patients (lower right panel).

(F) Heatmap of DEGs among fibroblast subclusters.

(G) UMAP plot showing the expression of three CAFs subpopulations markers (*PDGFRA*, *RGS5*, and *FGFBP2*).

(H) Gene set variation analysis highlighting the pathway enrichment across three

CAF subpopulations.

**Supplementary Fig. 2. Pseudotime analysis identifying a differentiation trajectory from HPCs to CAFs in human hepatocarcinogenesis**

(A) Pseudotime trajectories utilizing Monocle 3 showing the dynamic differentiation of HPCs into three CAFs subpopulations. Cells at the end of the branch are represented by dots of different colors.

(B) A heatmap ordered by pseudotime shows the dynamic expression changes of genes across the three CAF subclusters.

(C) Pseudotime trajectory plot showing expression of three CAFs subclusters markers (*PDGFRA*, *FGFBP2,* and *RGS5*).

**Supplementary Fig. 3. Different concentrations of GCDCA at different times on the differentiation of HPCs.**

1. Morphological changes in rat HPC line WB-F344 cultured with (1 μM, 10 μM, 100

μM) GCDCA for 0 days, 7 days and 14 days *in vitro*. Scale bar, 100 μm.

1. Schematic diagram of the isolation and organoid culture of primary HPCs from rat

liver after an 8-week DEN treatment.

**Supplementary Fig. 4. The expression of various cell markers in rat groups was detected by RT-PCR.**

(A, B) GCDCA (8 μmol/100 g, i.p.) was administered via intraperitoneal injection

continuously for 4weeks from week 8 to week 12. The control group received PBS. Real-time PCR analysis marker genes in each cell type: HPCs, myofibroblasts, hepatocytes, bile duct cells, endothelial cells, macrophage cells, T cells, B cells, DC cells, NK cells. Data are shown as the mean ± SD. **p*<0.05, ***p* < 0.01.

(C, D) Gene expression values (in FPKM) in GCDCA-treated rats. Data are shown as the mean ± SD. **p*<0.05, ***p* < 0.01.

**
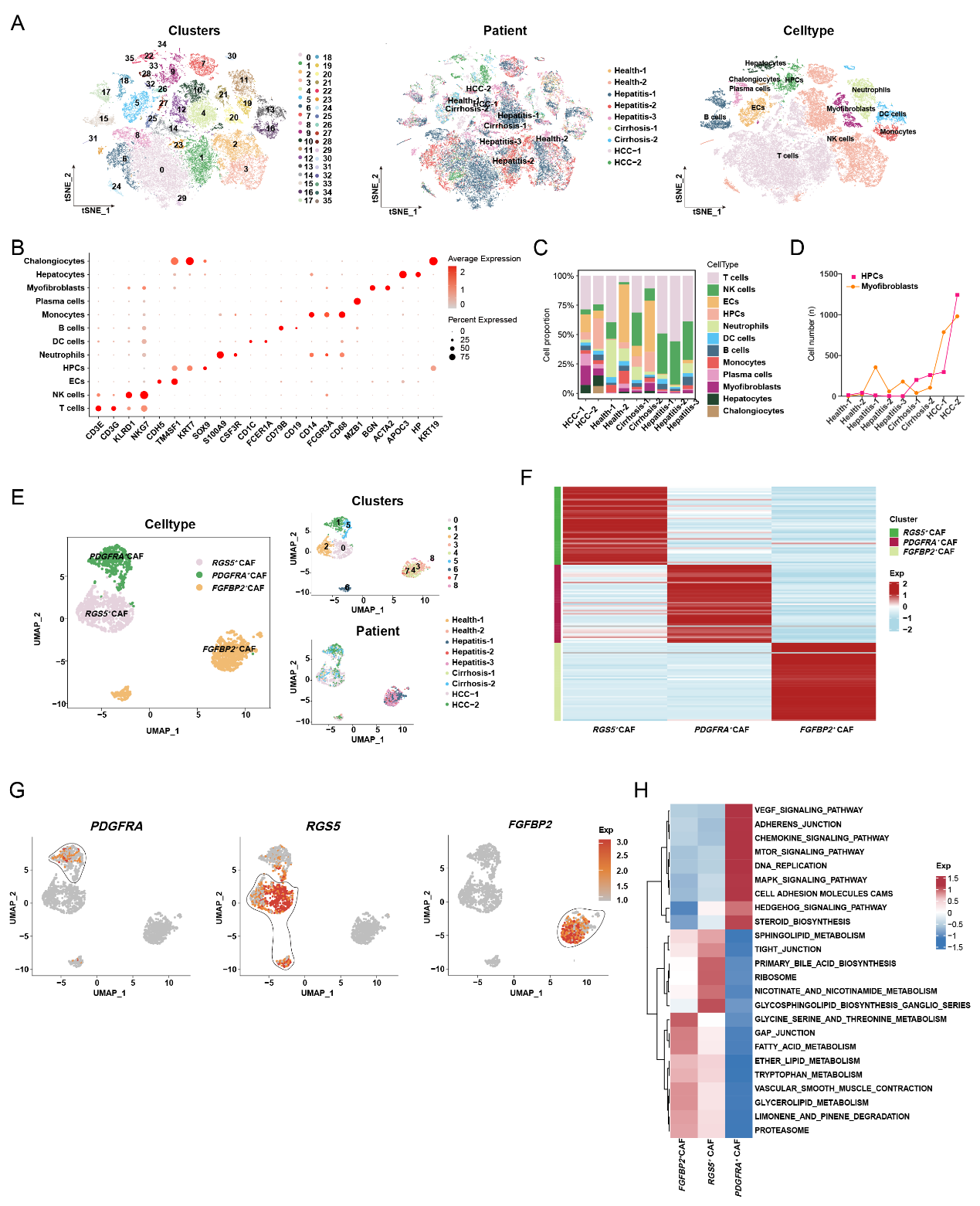
**

**Fig. S2**

**
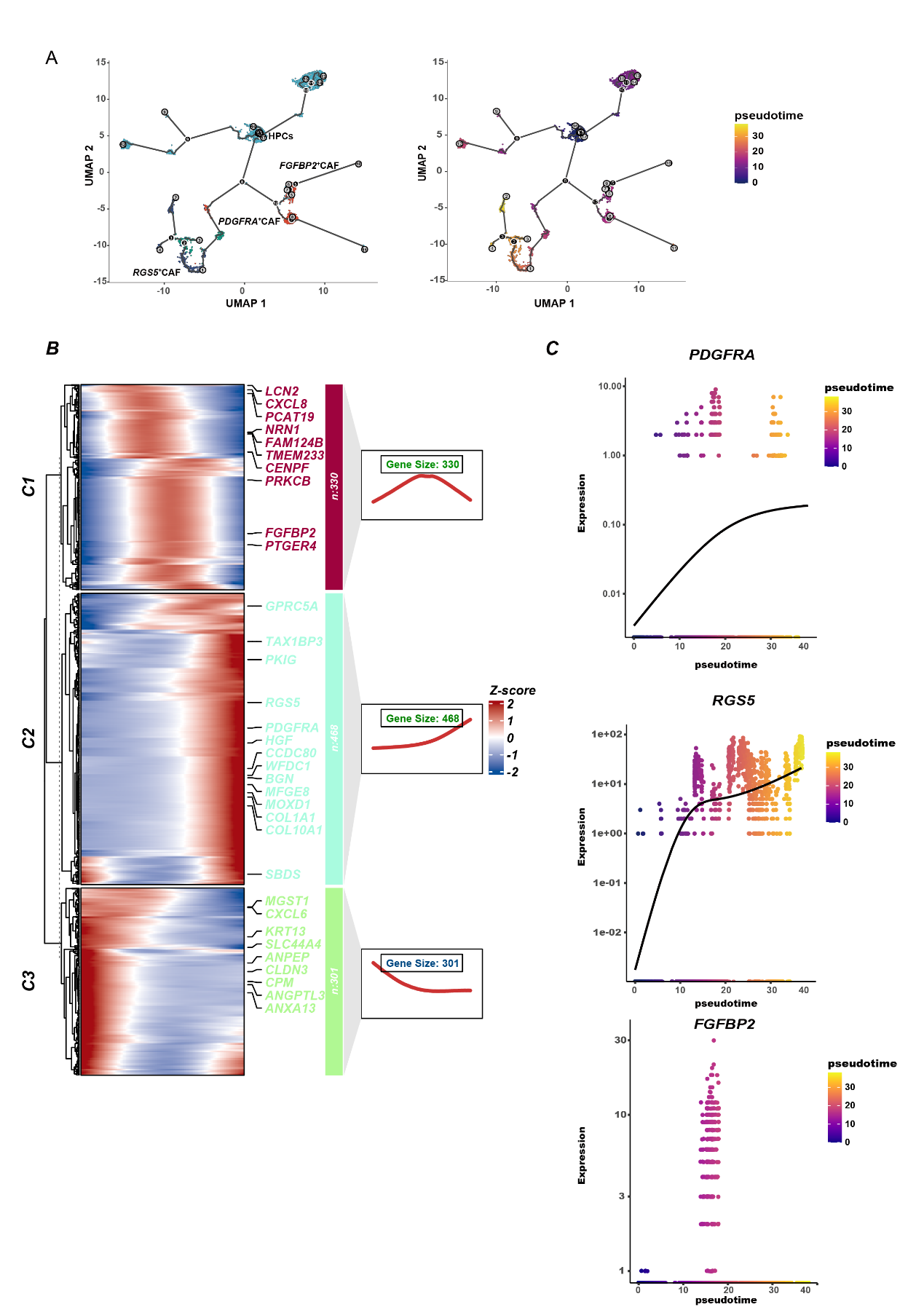
**

**Fig. S3**

**
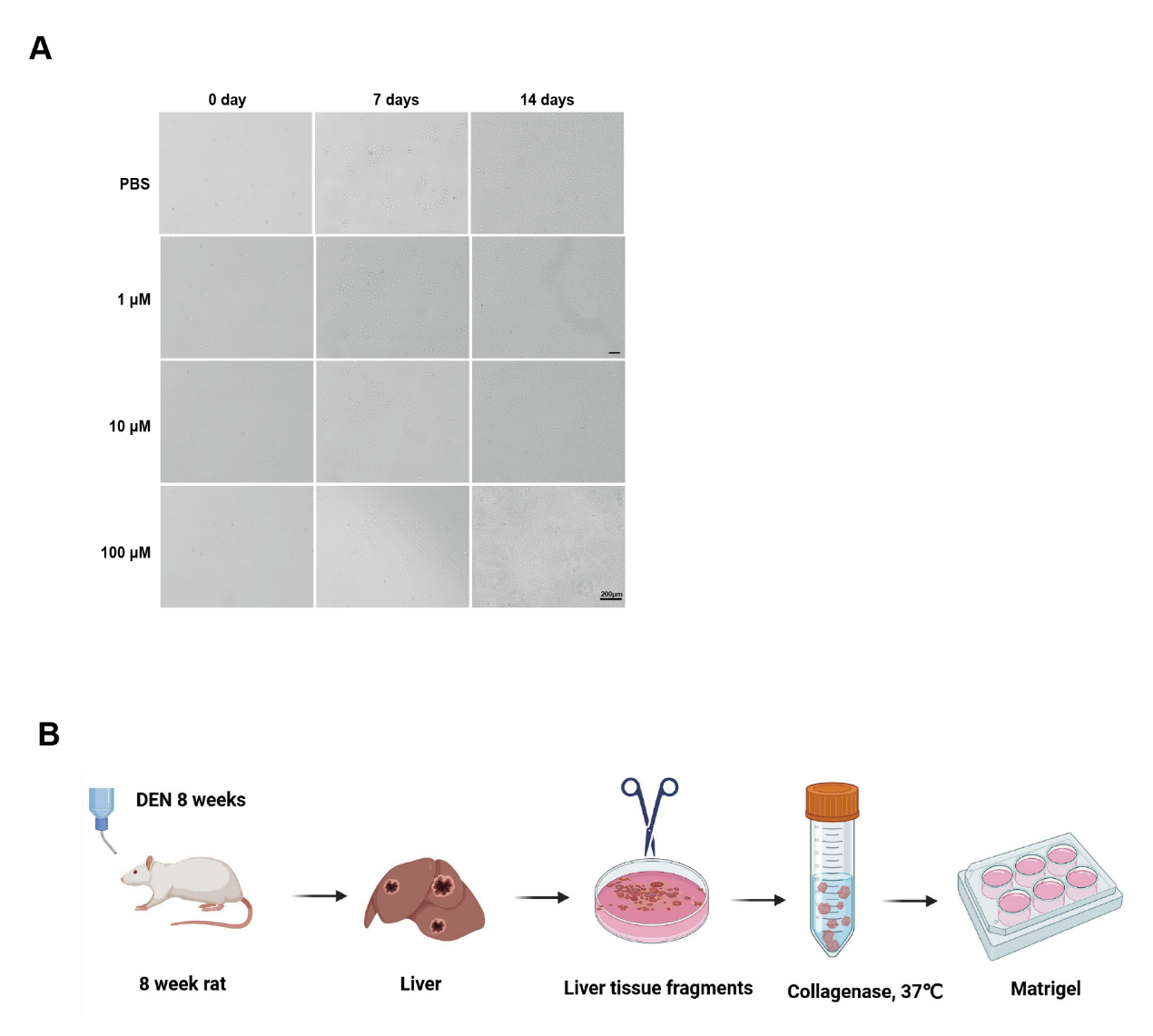
**

**Fig. S4**

**
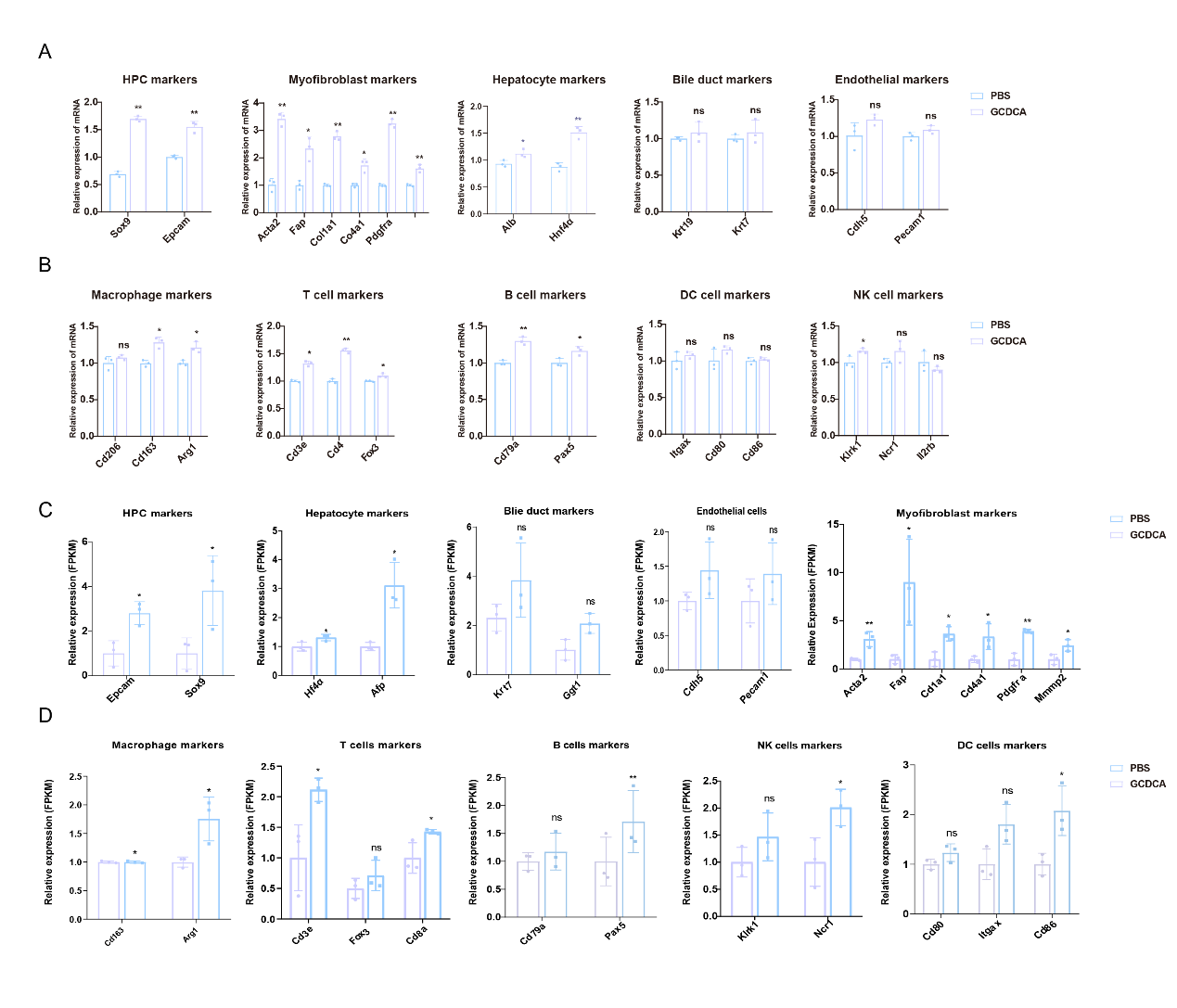
**
